# DNMT3a Downregulation Ttriggered Upregulation of GABA_A_ Receptor in the mPFC Promotes Paclitaxel‐Induced Pain and Anxiety in Male Mice

**DOI:** 10.1002/advs.202407387

**Published:** 2024-12-16

**Authors:** Lixia Tian, Xu‐Hui Li, Yu‐Long Zhao, Hui‐Yuan Yi, Xue‐Ru Liu, Rongrong Yao, Xue‐Mei Hou, Xuan Zhu, Fu‐Quan Huo, Tao Chen, Lingli Liang

**Affiliations:** ^1^ Department of Physiology and Pathophysiology School of Basic Medical Sciences Institute of Neuroscience Translational Medicine Institute Xi'an Jiaotong University Health Science Center Xi'an Shaanxi 710061 P. R. China; ^2^ Center for Neuron and Disease Frontier Institutes of Science and Technology Xi'an Jiaotong University Xi'an Shaanxi 710061 P. R. China; ^3^ Department of Anatomy and K.K. Leung Brain Research Centre Fourth Military Medical University Xi'an 710032 P. R. China

**Keywords:** anxiety, DNMT3a, GABA_A_ receptor, paclitaxel, neuropathic pain

## Abstract

Chemotherapeutic agents, such as paclitaxel (PTX), induce neuroplastic changes and alter gene expression in the prefrontal cortex (PFC), which may be associated with chemotherapy‐induced pain and negative emotions. Notably, DNA methylation undergoes adaptive changes in neurological disorders, emerging as a promising target for neuromodulation. In this study, systemic administration of PTX leads to a decrease in the expression of the DNA methyltransferase DNMT3a, while concurrently upregulating the expression of *Gabrb1* mRNA and its encoded GABA_A_Rβ1 protein in the medial PFC (mPFC) of male mice. Overexpression of DNMT3a in the mPFC alleviates PTX‐induced pain hypersensitivity, and anxiety‐like behavior in these mice. Additionally, it reverses the PTX‐induced increase in inhibitory synaptic transmission in the pyramidal neurons of the mPFC. Mechanistically, the upregulation of GABA_A_Rβ1 in the mPFC is linked to the reduced expression of DNMT3a and DNA hypomethylation at the promoter region of the *Gabrb1* gene. Furthermore, a long‐term diet rich in methyl donors alleviates PTX‐induced pain hypersensitivity and anxiety‐like behavior in mice. These findings suggest that the DNMT3a‐mediated upregulation of GABA_A_Rβ1 in the mPFC contributes to PTX‐induced neuropathic pain and anxiety, highlighting DNA methylation‐dependent epigenetic regulation as a potential therapeutic target for addressing chemotherapy‐induced cortical dysfunction.

## Graphical abstract

Paclitaxel induces DNMT3a‐dependent DNA hypomethylation in the mPFC, which contributes to paclitaxel‐induced hyperalgesia and anxiety by upregulating GABA_A_ receptors and enhancing inhibitory transmission in mPFC glutamatergic neurons. Strategies that increase DNA methylation, such as a long‐term methyl donor diet, may offer benefits for managing chemotherapy‐related pain and anxiety. Additionally, therapeutic drugs targeting GABA_A_ receptors hold promise as treatments for chemotherapy‐induced pain and anxiety.

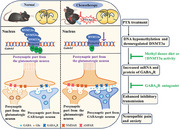

## 1 Introduction

Advancements in the development of novel chemotherapeutic drugs have significantly extended the survival of cancer patients.^[^ ^1^ ^]^ However, these advancements are often accompanied by a range of side effects.^[^ ^2^ ^]^ For example, chemotherapy drugs cause peripheral neuropathy, frequently leading to neuropathic pain—a prevalent neurological side effect.^[^ ^2a,b^ ^]^ Additionally, the neurotoxicity of these drugs on the central nervous system, commonly referred to as “chemobrain”, is associated with memory loss and cognitive impairment.^[^ ^2c^ ^]^ Chemotherapy and chronic pain are often accompanied by negative emotional reactions, such as anxiety and depression, which profoundly affect the quality of life for cancer patients during treatment.^[^ ^2d^ ^]^ However, the underlying mechanisms driving these adverse effects remain poorly understood, and effective therapeutic strategies are still lacking.

DNA methylation, a key epigenetic modification, plays an essential role in regulating gene expression.^[^ ^3^ ^]^ This process is primarily catalyzed by the DNA methyltransferase (DNMT) family—DNMT3a, DNMT3b, and DNMT1—which methylate cytosine residues at the fifth position to form 5‐methylcytosine (5‐mC).^[^ ^3^ ^]^ DNMT3a and DNMT3b function as de novo methyltransferases, establishing new methylation patterns, whereas DNMT1 mainly maintains existing methylation patterns across the genome.^[^ ^3b^ ^]^ Both DNA methylation and DNMTs have been implicated in a broad range of physiological and pathological processes,^[^ ^3^, ^4^ ^]^ and DNMTs have recently emerged as promising targets for cancer therapy.^[^ ^5^ ^]^ For example, it has been shown that DNMT3a in primary sensory neurons of the dorsal root ganglion is critical in neurogenesis associated with nerve injury,^[^ ^6^ ^]^ highlighting its potential as a therapeutic target for managing neuropathic pain. Prior research has also pointed to the potential toxicity of chemotherapy on the prefrontal cortex (PFC).^[^ ^7^ ^]^ Our findings revealed that systemic administration of paclitaxel (PTX), a taxane‐class chemotherapeutic agent, induced changes in the expression of several synaptic transmission‐related genes in the PFC, including *Syt9*, *Gabrb1*, *Gabrg1*, and *Drd1*.^[^ ^8^ ^]^ Given the PFC's crucial role in modulating both the sensory and emotional dimensions of chronic pain,^[^ ^9^ ^]^ it is imperative to understand the mechanisms, especially DNA methylation and DNMTs, underlying PFC dysfunction in the modulation of chemotherapy‐induced pain hypersensitivity and associated negative emotions.

In this study, we found that PTX treatment caused pain hypersensitivity and anxiety‐like behaviors in mice, along with a significant decrease in Fos protein expression in the medial PFC (mPFC). Specifically, PTX caused DNMT3a‐dependent DNA hypomethylation, leading to the upregulation of multiple GABA_A_ receptor subunits, particularly the β1 subunit. Restoring DNMT3a expression in the mPFC attenuated the PTX‐induced enhancement of inhibitory synaptic transmission, alleviating both pain hypersensitivity and anxiety‐like behaviors, and conversely, inhibiting DNMT3a exacerbated these effects. The overexpression of DNMT3a increased DNA methylation levels at the promoters of *Gabrb1* genes, leading to the silencing of their expression. Therefore, DNMT3a and GABA_A_ receptor β1 subunit represent promising therapeutic targets for managing chemotherapy‐induced neuropathic pain and anxiety. Additionally, our study provided an insightful solution by demonstrating that a long‐term methyl donor diet in mice can prevent chemotherapy‐induced pain hypersensitivity and anxiety.

## 2 Results

### 2.1 PTX‐Treated Mice Exhibit Pain Hypersensitivity and Emotional Distress, Along with Reduced Neuronal Activity in Specific Cortical Regions

A preclinical model of PTX‐induced neuropathic pain and negative emotional states has been previously established.^[^ ^8^, ^11^ ^]^ Consistent with these findings, our study shows that intraperitoneal administration of PTX (4 mg kg^−1^) led to pain hypersensitivity, indicated by an increased paw withdrawal frequency (PWF) to 0.07 and 0.4 g von Frey filaments and reduced paw withdrawal latency (PWL) to both heat and cold stimuli. These effects began on day 4 post‐injection, peaked between days 7 and 14, and partially resolved by day 21 (Figure S1A–D, Supporting Information). To confirm that these effects were not due to motor impairments from PTX toxicity, we evaluated locomotor function by assessing placing, grasping, and righting reflexes. No locomotor dysfunction was observed in PTX‐treated mice (Figure S1E, Supporting Information). In addition, the rotarod test showed that PTX‐treated mice had similar revolution speeds and retention times on the rods compared to control mice (Figure S1F,G, Supporting Information), suggesting that PTX induces pain hypersensitivity without impairing locomotion.

PTX treatment also induced anxiety‐like behaviors, as demonstrated in the open field test (OFT) and elevated plus maze test (EPMT) (Figure S1H–N, Supporting Information). Specifically, on day 21 post‐treatment, PTX‐treated mice spent significantly less time in the center area of the OFT compared to controls, though no difference was seen on days 7 and 14 (Figure S1H–J, Supporting Information). The total distance traveled in the OFT remained comparable between vehicle and PTX groups (Figure S1K, Supporting Information). In the EPMT on day 21, PTX‐treated mice showed fewer entries and a reduced percentage of time spent in the open arms relative to controls (Figure S1L–N, Supporting Information). Additionally, PTX treatment induced depressive‐like behavior, with the forced swim test (FST) revealing significantly increased immobility times in PTX‐treated mice on days 14 and 21 post‐treatment compared to controls (Figure S1O, Supporting Information).

**Figure 1:**
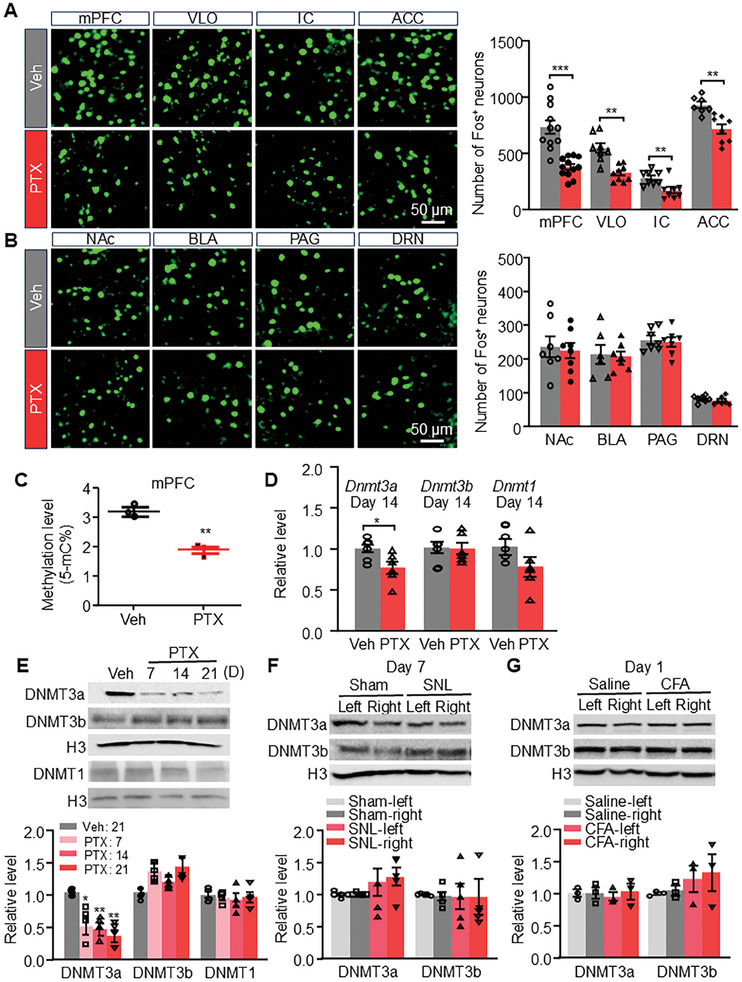
Paclitaxel (PTX) induces changes in the expression of Fos protein and DNMT3a in the mPFC of male mice. A) Expression of Fos protein in the mPFC, ventrolateral orbital cortex (VLO), insular cortex (IC) and anterior cingulate cortex (ACC) following treatment with PTX or vehicle (Veh). The histogram illustrates the quantification of Fos+ neurons in these brain regions. *n*  =  3.  **P < 0.01, ***P < 0.001, versus the Veh group, using two‐tailed unpaired *t*‐tests. B) Expression of Fos protein in the nucleus accumbens core (NAc), basolateral amygdala (BLA), periaqueductal gray (PAG), and dorsal raphe nucleus (DRN) following treatment with PTX or Veh. Scale bar: 50 µm. C) Methylation levels of 5‐methylcytosine (5‐mC) in the mPFC following treatment with PTX or Veh. D) Expression of *Dnmt3a*, *Dnmt3b*, and *Dnmt1* mRNAs in the mPFC following treatment with PTX or Veh. *n*  =  3‐4. *p < 0.05, **p < 0.01, versus the Veh group, using two‐tailed unpaired *t*‐tests. E) DNMT3a, DNMT3b, and DNMT1 proteins expression in the mPFC following treatment with PTX or Veh. *n*  =  4. *p < 0.05, **p < 0.01, versus the Veh group, using one‐way ANOVA followed by *post hoc* Tukey test. F) Expression of DNMT3a and DNMT3b proteins in the left and right sides of the mPFC following unilateral spinal nerve ligation (SNL) or sham surgery. *n*  =  5. G) Expression of DNMT3a and DNMT3b proteins in the left and right sides of the mPFC one day after intraplantar injection of physiological saline or complete Freund's adjuvant (CFA) in the left paw. *n*  =  3. Representative western blots (top panels) and a summary of densitometric analysis (bottom graphs).

Accumulating evidence suggests the involvement of cortical and subcortical areas in the processing of pain and emotional responses.^[^ ^9^, ^12^ ^]^ We utilized immunostaining for c‐Fos protein, an activity‐dependent neuronal marker,^[^ ^13^ ^]^ to explore potential alterations in brain regions associated with chemotherapy‐induced pain and related anxiety and depression. Our findings revealed that compared to the vehicle group, the number of Fos‐immunoreactive (Fos+) neurons was significantly reduced by 48.1%, 33.2%, 38.7%, and 11.9% in the medial prefrontal cortex (mPFC), ventrolateral orbital cortex (VLO), insular cortex (IC), and anterior cingulate cortex (ACC) respectively, in the PTX group (**Figure** **1**A; Figure S2A,B, Supporting Information). However, we observed no alterations in the expression of Fos protein in the nucleus accumbens core (NAc), basolateral amygdala (BLA), periaqueductal gray (PAG), and dorsal raphe nucleus (DRN), which are also implicated in the processing of sensory or emotional aspects of pain (Figure 1B; Figure S2B–D, Supporting Information).^[^ ^12b,c^ ^]^ These results indicate that cortical areas, particularly the mPFC, are extensively impacted by PTX treatment.

We also observed Fos expression in the spinal dorsal horn of PTX‐treated mice. Fos expression in this region is known to be induced by peripheral noxious or innocuous stimuli under chronic pain conditions.^[^ ^14^ ^]^ To induce acute Fos expression, a mechanical stimulus was applied to the left paw by brushing for 1 min, 2 h prior to perfusion. As shown in Figure S2E–H (Supporting Information), there was a significant increase in Fos+ neurons in both the superficial (laminae I‐III) and deeper (laminae IV‐VI) layers of the spinal dorsal horn. Compared to the vehicle group, the number of Fos+ neurons in the PTX group increased by 2.79‐fold on the left side and 2.90‐fold on the right side within the superficial laminae (Figure S2G, Supporting Information). Similarly, in the deeper laminae, Fos+ neuron numbers rose by 2.53‐fold on the left and 2.86‐fold on the right (Figure S2H, Supporting Information). Notably, in the PTX group, the left superficial dorsal horn had a greater number of Fos+ neurons than the right side, a pattern not seen in the control group (Figure S2G, Supporting Information). These findings suggest that PTX treatment significantly increases the activity of nociceptive neurons in the spinal dorsal horn, indicating that PTX‐treated mice experience a pathological state of pain hypersensitivity.

### 2.2 PTX Induces DNA Hypomethylation and Reduces DNMT3a Expression in the mPFC

To investigate the potential role of DNA methylation and DNA methyltransferases (DNMTs) in the mPFC regarding chemotherapy‐induced pain, anxiety, and depression, we first assessed DNA methylation patterns and DNMT expression in this preclinical chemotherapy model induced by PTX. Our findings indicate that PTX administration leads to global hypomethylation in the mPFC of male mice, as evidenced by a reduction in 5‐mC levels (Figure 1C). Furthermore, PTX treatment resulted in decreased expression of DNMT3a at both the mRNA (Figure 1D) and protein levels (Figure 1E), while DNMT3b and DNMT1 levels remained unchanged (Figure 1D,E). This was further validated with an additional DNMT3a antibody capable of detecting three variants of DNMT3a, confirming its downregulation following PTX treatment in male mice (Figure S3A, Supporting Information).

Additionally, we evaluated the expression of the three members of the ten‐eleven translocation (TET) family of methylcytosine dioxygenases—TET1, TET2, and TET3—in the mPFC of male mice after PTX injection. The TET enzymes are involved in DNA demethylation, catalyzing the oxidation of 5‐mC to 5‐hydroxymethylcytosine (5‐hmC), which promotes DNA demethylation and subsequent gene activation.^[^ ^15^ ^]^ Notably, PTX treatment induced a time‐dependent reduction in the expression of TET1 and TET2, with no effect observed on TET3 expression (Figure S3B–D, Supporting Information). Given that PTX decreased total DNA methylation in the mPFC, the suppression of *Dnmt3a* mRNA and the resulting decrease in DNMT3a protein likely represent the primary mechanisms driving PTX‐induced hypomethylation in this region.

To explore the specificity or generality of PTX‐induced DNA hypomethylation and downregulation of DNMT3a expression across various chronic pain conditions, we evaluated changes in DNMT3a expression in the mPFC using additional preclinical chronic pain models. Notably, oxaliplatin (OXA), a widely used platinum‐based chemotherapy agent, similarly reduced DNMT3a protein expression in male mice (Figure S3E, Supporting Information). While previous research has demonstrated hypomethylation and reduced DNMT expression in the mPFC 6 months post‐partial sciatic nerve ligation surgery,^[^ ^16^ ^]^ we aimed to assess whether peripheral nerve injury or chronic inflammation affected DNMT3a expression in the mPFC at earlier time points. However, we did not observe significant changes in DNMT3a or DNMT3b protein levels in the mPFC 7 days following lumbar 4 spinal nerve ligation (SNL) (Figure 1F) or one day after complete Freund's adjuvant (CFA) injection (Figure 1G). Additionally, there was no change in DNMT3a expression in the mPFC of female mice (Figure S3F, Supporting Information). These findings suggest that the downregulation of DNMT3a in the mPFC induced by PTX is specific to male mice.

### 2.3 Overexpression of DNMT3a Alleviates Pain Hypersensitivity and Anxiety‐Like Behavior Induced by PTX

**Figure 2:**
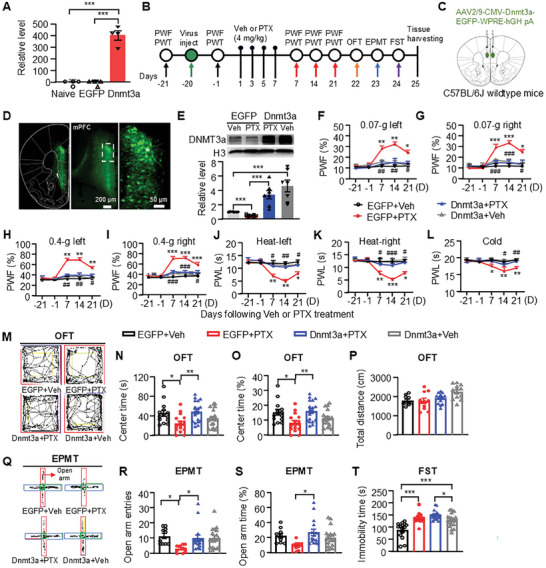
Overexpression of DNMT3a in the mPFC alleviates pain hypersensitivity and anxiety‐like behavior induced by paclitaxel (PTX). A) The level of *Dnmt3a* mRNA in cultured cerebral cortex 2 days post‐transduction with AAV‐*Dnmt3a* or AAV‐EGFP. *n*  = 4. ***P < 0.001, using one‐way ANOVA followed by *post hoc* Tukey test. B) A schematic representation of the virus injection, drug administration, and behavioral tests. Intraperitoneal injections of PTX or vehicle (Veh) were performed three weeks after the microinjection of AAV‐EGFP or AAV‐*Dnmt3a* into the mPFC. C) A schematic diagram illustrating the virus injection into the mPFC. D) EGFP expression in the mPFC of mice following AAV‐CMV‐EGFP injection. Scale bar: 200 µm; 50 µm. E) DNMT3a protein expression levels in the mPFC at the endpoint of the experiment. Representative western blots (top panels) and a summary of densitometric analysis (bottom graphs). *n*  = 7. ***p < 0.001, using two‐tailed unpaired *t*‐test or one‐way ANOVA followed by *post hoc* Tukey test. F‐L) Paw withdrawal responses to 0.07 g *von* Frey filament (F and G), 0.4 g *von* Frey filament (H and I), heat (J and K) and cold stimulation (L). *n*  = 10–12. *p < 0.05, **p < 0.01, ***p < 0.001, versus the AAV‐EGFP plus vehicle‐treated group (EGFP + Veh); ^#^ *p* < 0.05, ^##^ *p* < 0.01, ^###^ *p* < 0.001, versus the AAV‐EGFP plus PTX‐treated group (EGFP + PTX), using two‐way ANOVA followed by *post hoc* Tukey test. M‐P) Schematic traces of the open field test (OFT) (M) and the time spent in the center (N) and the percentage of time spent in the center (O), and the total distance traveled in the center (P) in the OFT. Q‐S) Schematic traces of the elevated plus‐maze test (EPMT) (Q) and the number of entries into the open arms (R) and the percentage of time (S) spent in the open arms in the EPMT. T) The immobility time in the forced swim test (FST). *n*  = 10–21. *p < 0.05, **p < 0.01, ***p < 0.001, using one‐way ANOVA followed by *post hoc* Tukey test. EGFP: AAV‐EGFP; Dnmt3a: AAV‐*Dnmt3a*.

To investigate the relationship between the downregulation of DNMT3a expression in the mPFC induced by PTX and chemotherapy‐related pain and negative emotions, we aimed to determine whether restoring DNMT3a levels in the mPFC could alleviate pain hypersensitivity, as well as anxiety‐like and depression‐like behaviors in PTX‐treated mice. We constructed an adeno‐associated viral (AAV) vector expressing full‐length *Dnmt3a* mRNA (AAV‐*Dnmt3a*) and confirmed successful overexpression in cultured cortical neurons compared to a control AAV vector expressing AAV‐CMV‐enhanced green fluorescent protein (AAV‐EGFP) (**Figure** **2**A). Three weeks prior to the intraperitoneal injection of PTX, we microinjected AAV‐*Dnmt3a* into both sides of the mPFC (Figure 2B–D). Three weeks post‐PTX injection, we observed a significant reduction in DNMT3a protein levels in the mPFC of the PTX‐treated group infected with AAV‐EGFP (EGFP + PTX) compared to the vehicle‐treated group infected with AAV‐EGFP (EGFP + vehicle) (Figure 2E). In contrast, infection with AAV‐*Dnmt3a* resulted in significant overexpression of DNMT3a protein in both PTX‐treated (Dnmt3a + PTX) and vehicle‐treated (Dnmt3a + vehicle) groups (Figure 2E).

Behaviorally, infection with AAV‐*Dnmt3a* significantly reduced mechanical allodynia and heat/cold hyperalgesia on days 7, 14, and 21 following PTX treatment (Figure 2F–L). Given that anxiety‐like and depression‐like behaviors typically emerge three weeks post‐PTX treatment, we conducted the OFT, EPMT, and FST on days 22, 23, and 24 in mice infected with AAV‐*Dnmt3a* (Figure 2M–T). Our results showed that DNMT3a overexpression significantly increased the percentage of time spent in the central area of the OFT (Figure 2M–O) without affecting motor function (Figure 2P). Furthermore, it significantly increased both the number of entries into the open arms and the percentage of time spent there in the EPMT (Figure 2Q–S), indicating that DNMT3a overexpression effectively mitigated PTX‐induced anxiety‐like behaviors. However, we observed no significant change in depression‐like behavior, as indicated by comparable immobility times between the Dnmt3a + vehicle and Dnmt3a + PTX groups in the FST (Figure 2T). Collectively, these findings suggest that downregulation of DNMT3a in the mPFC is crucial in the development of PTX‐mediated pain hypersensitivity and anxiety.

Conversely, a previous study demonstrated that knockdown of DNMT3a in the mPFC produces an anxiogenic effect in mice.^[^ ^17^ ^]^ In our investigation, we also examined the effects of DNMT3a knockdown in the mPFC by locally administering *Dnmt3a* siRNA into this brain region. Specifically, 500 ng of Dnmt3a siRNA was injected bilaterally every other day for a total of three injections to ensure sustained knockdown efficiency (Figure S4A, Supporting Information). Western blot analysis revealed a 26% reduction in DNMT3a protein levels in the mPFC following *Dnmt3a* siRNA administration (Figure S4B, Supporting Information). Behavioral assessments indicated that DNMT3a knockdown resulted in mechanical allodynia, thermal hyperalgesia, and cold hyperalgesia in pain‐related behavioral tests (Figure S4C–I, Supporting Information). Additionally, anxiety‐like behaviors were observed, as evidenced by the EPMT (Figure S4J–L, Supporting Information), while no such behaviors were noted in the OFT (Figure S4M–P, Supporting Information). Consistently, DNMT3a does not appear to be associated with depressive behavior, as immobility times in the FST were comparable among the groups (Figure S4Q, Supporting Information).

### 2.4 Overexpression of DNMT3a in Pyramidal Neurons of the mPFC Attenuates Neuropathic Pain and Anxiety‐Like Behavior Induced by PTX

**Figure 3:**
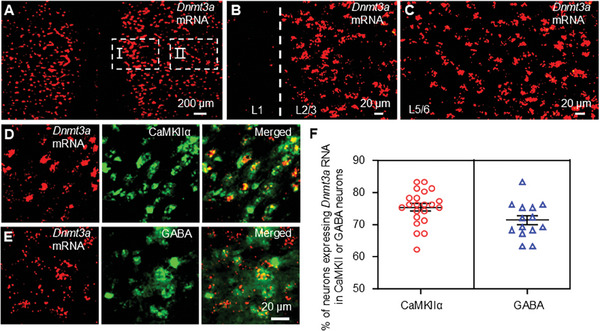
DNMT3a is expressed nonselectively in the mPFC. A) RNAScope in situ hybridization (ISH) showing *Dnmt3a* mRNA expression in the mPFC of naive mice. Scale bar: 200 µm. B) *Dnmt3a* mRNA expression in layer 1 (L1) and layers 2/3 (L2/3) of the mPFC (Magnification of region I in Figure A). Scale bar: 20 µm. C) *Dnmt3a* mRNA expression in layers 5/6 (L5/6) of the mPFC (Magnification of region II in Figure A). Scale bar: 20 µm. D‐E) Colocalization of *Dnmt3a* mRNA with CaMKIIα (D) and GABA (E) in the mPFC, assessed using ISH and immunofluorescence. Scale bar: 20 µm. F) Percentage of neurons expressing *Dnmt3a* mRNA among CaMKIIα‐positive and GABA‐positive neurons. n = 3.

The two primary types of neurons in the mPFC—pyramidal neurons and GABAergic inhibitory neurons—play distinct roles in pain modulation.^[^ ^18^ ^]^ To elucidate the specific function of DNMT3a within the mPFC, we employed RNAscope technology in conjunction with immunofluorescence to examine the distribution of *Dnmt3a* mRNA in mPFC neurons. Our findings showed that *Dnmt3a* mRNA was prominently expressed as distinct puncta across layers 2/3 to 5/6 of the mPFC (**Figure** **3**A–C). Notably, 75.1% of CaMKIIα‐positive pyramidal neurons and 71.1% of GABA‐positive inhibitory neurons displayed *Dnmt3a* mRNA expression (Figure 3D–F). Thus, DNMT3a is expressed in both excitatory and inhibitory neurons within the mPFC, indicating a non‐selective pattern of expression.

**Figure 4:**
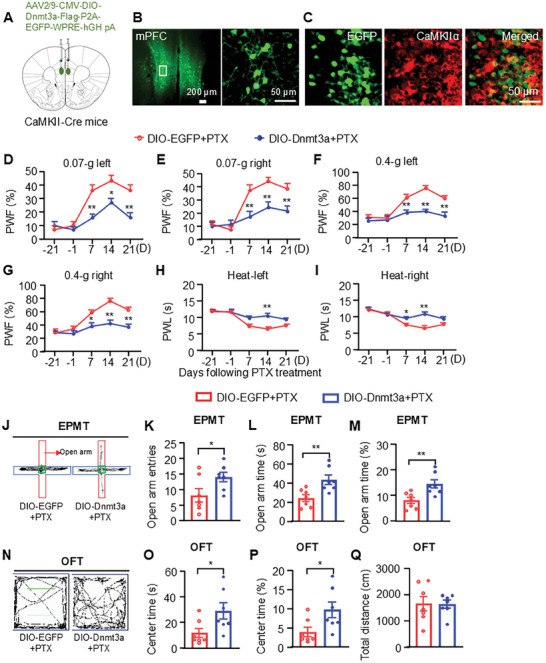
Overexpression of DNMT3a in pyramidal neurons of the mPFC alleviates pain hypersensitivity and anxiety‐like behavior induced by paclitaxel (PTX). A) Schematic diagram of mPFC injected with AAV‐DIO‐*Dnmt3a* in CaMKII‐Cre mice. B) Expression of EGFP in the mPFC of CaMKII‐Cre mice following AAV‐DIO‐*Dnmt3a* injection. Scale bar: 200 µm; 50 µm. C) Colocalization of EGFP (green) and CaMKIIα (red) in the mPFC across three mice. Scale bar: 50 µm. D‐I) PTX was administered three weeks after the microinjection of AAV‐DIO‐EGFP or AAV‐DIO‐*Dnmt3a* into the mPFC, after which behavioral testing was conducted. Paw withdrawal responses to 0.07 g *von* Frey filament (D, E), 0.4 g *von* Frey filament (F, G), and heat stimulation (H, I) following treatment with PTX. *n*  = 7. *p < 0.05, **p < 0.01, versus the AAV‐DIO‐EGFP plus PTX‐treated (DIO‐EGFP + PTX) group, using two‐way ANOVA followed by *post hoc* Tukey test. J‐M) Schematic traces of the elevated plus‐maze test (EPMT) (J) and the number of entries into the open arms (K), the time (L), and the percentage of time (M) spent in the open arms in the EPMT. N‐Q) Schematic traces of the open field test (OFT) (N) and the time spent in the center (O) and the percentage of time spent in the center (P), and the total distance traveled in the center (Q) in the OFT. *n*  = 7. *p < 0.05, **p < 0.01, versus the DIO‐EGFP + PTX group at the corresponding time points, using two‐tailed unpaired *t*‐tests.

To investigate the distinct roles of DNMT3a in pyramidal versus GABAergic neurons, we selectively overexpressed DNMT3a in these neuronal populations by injecting AAV2/9‐DIO‐*Dnmt3a* or a control virus (AAV2/9‐DIO‐EGFP) into the mPFC of CaMKII‐Cre or GAD‐Cre mice (**Figure** **4**A–C; Figure S5A,B, Supporting Information). Pain behavioral assessments revealed that specific overexpression of DNMT3a in pyramidal neurons, but not in GABAergic neurons, significantly alleviated PTX‐induced mechanical allodynia and heat hyperalgesia on days 7, 14, and 21 post‐PTX treatment (Figure 4D–I; Figure S5C–H, Supporting Information). Furthermore, targeted overexpression of DNMT3a in pyramidal neurons, but not in GABAergic neurons, was associated with an increased frequency of open arm entries and a higher percentage of time spent in the open arms during the EPMT in PTX‐treated mice (Figure 4J–M; Figure S5I–L, Supporting Information). Additionally, on days 22 and 23 post‐PTX treatment, mice with specific overexpression of DNMT3a in pyramidal neurons, but not in GABAergic neurons, spent a significantly greater percentage of time in the center of the OFT compared to the control group (Figure 4N–P; Figure S5M–O, Supporting Information). Consistent with our earlier findings, DNMT3a overexpression in either pyramidal neurons or GABAergic neurons did not alter the total distance traveled in the OFT in PTX‐treated mice (Figure 4Q; Figure S5P, Supporting Information). Collectively, these results underscore the critical role of DNMT3a in pyramidal neurons for modulating PTX‐induced pain hypersensitivity and anxiety.

### 2.5 Overexpression of DNMT3a Reverses the PTX‐induced Increase in Inhibitory Synaptic Transmission in Pyramidal Neurons of the mPFC

**Figure 5:**
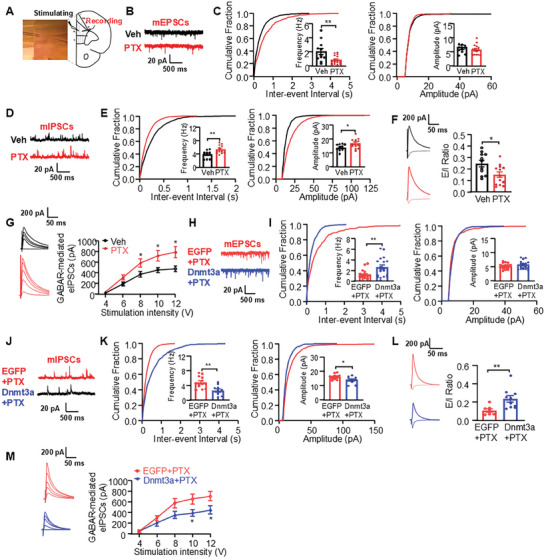
Overexpression of DNMT3a reverses the increased inhibitory synaptic transmission in the mPFC following paclitaxel (PTX) injection. A) Representative recording diagram and photograph showing the placement of stimulating electrode in layer II/III and recording pipette in layer V of mPFC. B) Representative mEPSCs traces in vehicle (Veh)‐ and PTX‐treated groups. C) Cumulative interevent interval (left) and amplitude histograms (right) of the mEPSCs. Statistical results show a reduction in frequency, but not amplitude, of the mEPSCs in the PTX‐treated group. Veh: *n* = 11 neurons/3 mice; PTX: *n* = 16 neurons/3 mice. D) Representative mIPSCs traces in Veh‐ and PTX‐treated groups. E) Cumulative interevent interval (left) and amplitude histograms (right) of the mIPSCs. Statistical results show an increase in both the frequency and amplitude of mIPSCs in the PTX‐treated group. Veh: *n* = 11 neurons/3 mice; PTX: *n* = 13 neurons/3 mice. F) The decreased E/I ratio in PTX‐treated mice. Veh: *n* = 11 neurons/4 mice; PTX: *n* = 13 neurons/5 mice. *p < 0.05, **p < 0.01, versus the Veh group, using two‐tailed unpaired *t*‐tests (C, E, F). G) The enhanced GABAR‐mediated eIPSCs in PTX‐treated mice. Veh: *n* = 12 neurons/5 mice; PTX: *n* = 13 neurons/7 mice. *p < 0.05, versus the Veh‐treated group, using two‐way ANOVA followed by *post hoc* Tukey test. H) Representative mEPSCs traces in the AAV‐EGFP plus PTX‐treated (EGFP + PTX) and the AAV‐*Dnmt3a* plus PTX‐treated (Dnmt3a + PTX) groups. I) Cumulative interevent interval (left) and amplitude histograms (right) of the mEPSCs. The statistical results show an increase in the frequency of mEPSCs, but not in their amplitude, in the Dnmt3a + PTX group. EGFP + PTX: *n* = 18 neurons/4 mice; Dnmt3a + PTX: *n* = 18 neurons/4 mice. J) Representative mIPSCs traces in the EGFP + PTX and Dnmt3a + PTX groups. K) Cumulative interevent interval (left) and amplitude histograms (right) of the mIPSCs. The statistical results show a decrease in both the frequency and amplitude of mIPSCs in the Dnmt3a + PTX group. EGFP + PTX: *n* = 13 neurons/5 mice; Dnmt3a + PTX: *n* = 11 neurons/4 mice. L) The increased E/I ratio in the Dnmt3a + PTX group. EGFP + PTX: *n* = 8 neurons/3 mice; Dnmt3a + PTX: *n* = 10 neurons/4 mice. **p < 0.01, versus the EGFP + PTX group, using two‐tailed unpaired *t*‐tests (I, K, L). M) The decreased GABAR‐mediated eIPSCs in the Dnmt3a + PTX group. EGFP + PTX: *n* = 8 neurons/3 mice; Dnmt3a + PTX: *n* = 9 neurons/3 mice. *p < 0.05, versus the EGFP + PTX group, using two‐way ANOVA followed by *post hoc* Tukey test.

To explore whether PTX changed the synaptic transmission in the mPFC, whole‐cell patch‐clamp recordings were performed on pyramidal neurons in the mPFC (**Figure** **5**A). At first, we recorded miniature excitatory postsynaptic currents (mEPSCs) and inhibitory postsynaptic currents (mIPSCs) in PTX‐ and vehicle‐treated mice. We found that PTX significantly decreased the frequency but not the amplitude of mEPSCs (Figure 5B,C). Conversely, PTX increased both the frequency and amplitude of mIPSCs (Figure 5D,E), suggesting a changed excitatory/inhibitory balance. We then recorded the ratio of evoked EPSC/IPSC (E/I ratio) in layer 5 pyramidal neurons by electrical stimulating the layers 2/3 of the mPFC. We found that the E/I ratio of pyramidal neurons was decreased in PTX‐treated mice compared with vehicle‐treated mice (Figure 5F). This was largely due to the enhanced GABA_A_ receptor (GABA_A_R)‐mediated synaptic responses, as demonstrated by that the evoked IPSCs were potentiated in PTX‐treated group (Figure 5G). Collectively, these results indicate that inhibitory synaptic transmission is elevated in the mPFC of mice following PTX treatment.

We then evaluated whether DNMT3a overexpression could reverse the increased inhibitory transmission observed in the mPFC of PTX‐treated mice. We found that overexpression of DNMT3a significantly elevated the frequency of mEPSCs while reducing both the frequency and amplitude of mIPSCs (Figure 5H–K). Additionally, DNMT3a overexpression led to an increased E/I ratio (Figure 5L) and diminished GABA_A_R‐mediated eIPSCs (Figure 5M) in PTX‐treated mice. These results suggest that the PTX‐induced reduction of DNMT3a contributes to the enhancement of GABA_A_R‐mediated inhibitory synaptic transmission in the mPFC.

### 2.6 The GABA_A_ Receptor Subunits in the mPFC Play a Role in Mediating PTX‐Induced Pain Hypersensitivity and Anxiety‐Like Behavior

GABA_A_R subtypes, composed of 19 distinct subunit genes, are localized to specific subcellular sites to enhance inhibitory neurotransmission.^[^ ^19^ ^]^ We hypothesize that GABA_A_R may mediate the effects of DNMT3a on PTX‐induced pain hypersensitivity and anxiety‐like behavior. To test this hypothesis, we assessed the expression levels of several key GABA_A_R subunits and explored their potential contribution to PTX‐induced pain hypersensitivity and anxiety‐like behavior.

Our previous research utilizing RNA high‐throughput sequencing and qPCR experiments indicated that PTX treatment increased GABA_A_R mRNA levels in the mPFC.^[^ ^8^ ^]^ In the present study, we further examined the protein expression of GABA_A_R subunits in the mPFC following PTX administration. Western blot analysis revealed significant elevations in the α1‐6, β1‐2, and γ1‐2 subunits of GABA_A_R from days 7 to 21 post‐PTX treatment (Figure S6A–E, Supporting Information). While the critical role of GABA_A_ receptors in the mPFC has been established in the context of neuropathic pain and anxiety‐like behavior,^[^ ^20^ ^]^ we sought to investigate the potential involvement of these upregulated GABA_A_R subunits in PTX‐induced pain hypersensitivity and anxiety‐like behavior. To explore this, we locally administered the GABA_A_R antagonist bicuculline (100 ng) into the mPFC (Figure S7A,B, Supporting Information). A single acute dose of bicuculline significantly alleviated mechanical allodynia in PTX‐treated mice (Figure S7C–F, Supporting Information). Moreover, repeated administrations of bicuculline produced a prolonged anti‐nociceptive effect on PTX‐induced mechanical allodynia and heat hyperalgesia, with analgesic effects lasting even 48 h after the final bicuculline injection (Figure S7G–L, Supporting Information). Additionally, bicuculline demonstrated anxiolytic effects in the EPMT (Figure S7M‐N), although similar effects were not observed in the OFT (Figure S7O–P, Supporting Information), and it did not affect the total distance traveled (Figure S7Q, Supporting Information).

### 2.7 Decreased Expression of DNMT3a Leads to the Upregulation of GABA_A_ Receptors in the mPFC, Contributing to PTX‐Induced Pain Hypersensitivity and Anxiety‐Like Behavior

**Figure 6:**
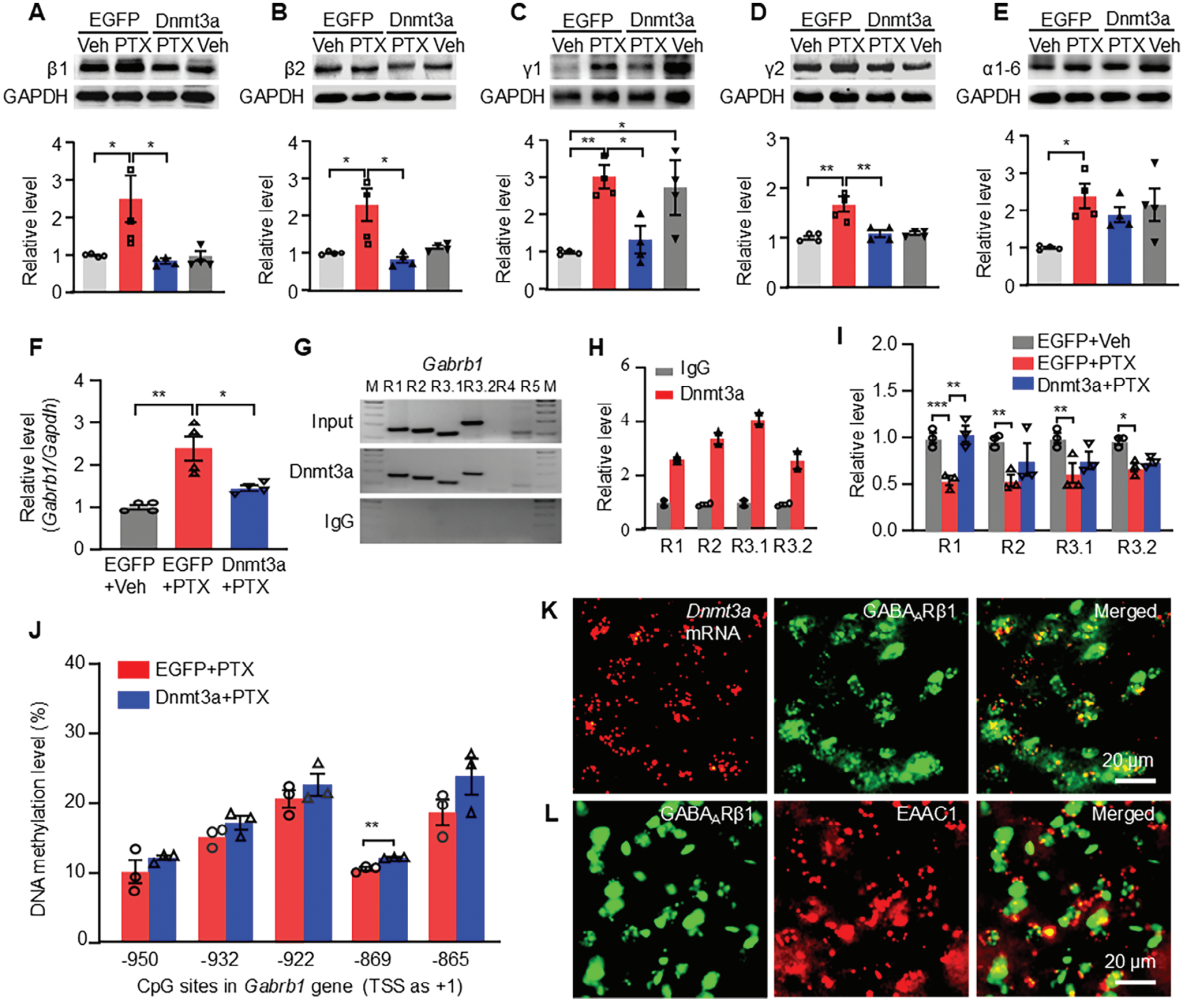
DNMT3a regulates DNA methylation of the *Gabrb1* gene and modulates GABA_A_ receptor subunit expression in the mPFC. A‐E) Expression levels of GABA_A_ receptor subunits in the mPFC following treatment with paclitaxel (PTX) or vehicle (Veh). β1 subunit (A), β2 subunit (B), γ1 subunit (C), γ2 subunit (D) and α1‐6 subunits (E). Representative western blots (top panels) and a summary of densitometric analysis (bottom graphs). *n* = 4–8. *p < 0.05, **P < 0.01, using one‐way ANOVA followed by *post hoc* Tukey test. F) Levels of *Gabrb1* mRNA in the mPFC following treatment with PTX or Veh. *n* = 4. *p < 0.05, **p < 0.01, using one‐way ANOVA followed by *post hoc* Tukey test. G‐H) Four regions (R1, ‐1002 bp to ‐845 bp; R2, ‐865 bp to ‐723 bp; R3.1, ‐209 bp to ‐98 bp; R3.2, ‐209 bp to ‐21 bp), but not other fragments (R4, ‐118 bp to +188 bp; R5, ‐38 bp to +188 bp), from the promoter and 5’‐end untranslated regions of the *Gabrb1* gene were immunoprecipitated by the rabbit anti‐DNMT3a (not by rabbit normal IgG in mouse mPFC). Input, total purified fragments. IgG, negative control. M, ladder marker. *n* = 2 biological replicates/6 mice/group. I) Binding activity of DNMT3a to regions within the *Gabrb1* gene. *n* = 3 biological replicates/9 mice/group. *p < 0.05, **p < 0.01, ***P < 0.001, using one‐way ANOVA followed by *post hoc* Tukey test. J) DNA methylation levels at CpG sites ‐950, ‐932, ‐922, ‐869, and ‐865 within the mPFC, as measured by bisulfite sequencing assay. *n* = 3 biological replicates/9 mice/group. **p < 0.01, versus the EGFP + PTX group, using two‐tailed unpaired *t*‐tests. K) Colocalization of *Dnmt3a* mRNA (red) with GABA_A_Rβ1 (green) in the mPFC. L) Colocalization of GABA_A_Rβ1 (green) with EAAC1 (red) in the mPFC. Scale bar: 20 µm. EGFP: AAV‐EGFP. Dnmt3a: AAV‐*Dnmt3a*.

We subsequently examined whether DNMT3a‐dependent DNA methylation regulates the transcription of GABA_A_R subunit genes and whether this mechanism contributes to the pathogenesis of PTX‐induced neuropathic pain and anxiety. To investigate this, we assessed the impact of DNMT3a overexpression on the protein levels of GABA_A_R subunits by locally administering AAV‐*Dnmt3a* into the mPFC. The results demonstrated that transfection with AAV‐*Dnmt3a* completely reversed the PTX‐induced upregulation of GABA_A_R subunit proteins (β1, β2, γ1, γ2), while exerting minimal effects on the expression of α1‐6 subunits (**Figure** **6**A–E). Notably, we observed that DNMT3a overexpression alone increased the expression of the γ1 subunit, without affecting any other subunits (Figure 6A–E). These findings suggest that DNMT3a plays a regulatory role in the expression of multiple GABA_A_R subunits in the mPFC.

We observed a significant increase in the levels of *Gabrb1* and *Gabrg1* mRNA in the mPFC of the EGFP + PTX group compared to the EGFP + vehicle group (Figure 6F; Figure S8A, Supporting Information). Remarkably, this increase was fully abolished by DNMT3a overexpression via AAV‐*Dnmt3a* microinjection into the mPFC (Figure 6F; Figure S8A, Supporting Information). To further investigate DNMT3a's regulatory effect on *Gabrb1* gene expression, we performed a ChIP assay and identified four distinct regions (R1: −1002 bp to −845 bp; R2: −865 bp to −723 bp; R3.1: −209 bp to −98 bp; R3.2: −209 bp to −21 bp) within the *Gabrb1* gene promoter that bind DNMT3a. This binding was confirmed by amplifying these regions from complexes immunoprecipitated with a DNMT3a antibody in nuclear fractions isolated from the mPFC of naive mice (Figure 6G,H). Notably, DNMT3a binding to these four regions was reduced in the EGFP + PTX group 24 days after PTX treatment compared to the EGFP + vehicle group (Figure 6I). However, DNMT3a overexpression selectively restored binding activity to the R1 region (−1002 bp to −845 bp) (Figure 6I). To pinpoint DNA methylation sites within the R1 region of the *Gabrb1* promoter, we used pyrosequencing, and our bisulfite sequencing assay revealed that DNMT3a overexpression significantly increased methylation at the ‐869 CpG site within the R1 region, which includes five CpG sites (Figure 6J). Additionally, RNAscope with a *Dnmt3a* probe and subsequent immunofluorescence for GABA_A_Rβ1 confirmed colocalization of *Dnmt3a* mRNA with GABA_A_Rβ1 in the mPFC (Figure 6K). Dual immunofluorescent staining further showed colocalization of GABA_A_Rβ1 with excitatory Amino Acid Carrier 1 (EAAC1), an excitatory neuron marker (Figure 6L). Collectively, this evidence indicates that GABA_A_Rβ1 is subject to DNMT3a‐dependent DNA methylation, regulating its expression in mPFC pyramidal neurons.

We further examined DNMT3a binding to the *Gabrg1* gene promoter and identified seven distinct binding regions (R1, −1002 bp to −789 bp; R2, −810 bp to −577 bp; R3, −598 bp to −381 bp; R4, −402 bp to −113 bp; R5, −132 bp to 100 bp; R6, 82 bp to 266 bp; R7, 247 bp to 472 bp), confirmed through amplification of these regions (Figure S8B,C, Supporting Information). Notably, PTX treatment reduced DNMT3a binding in four of these regions, while DNMT3a overexpression restored binding specifically to two regions (R1, −1002 bp to −789 bp; R3, −598 bp to −381 bp) (Figure S8D, Supporting Information).

These findings suggest that DNMT3a regulates the expression of both the *Gabrb1* and *Gabrg1* genes by binding to specific regions of their promoters, which in turn increases DNA methylation levels in those regions. Therefore, our study provides strong evidence for the role of DNMT3a‐mediated DNA methylation in controlling the expression of GABA_A_R subunits in the mPFC.

### 2.8 A Prolonged Methyl Donor Diet Mitigates PTX‐Induced Pain Hypersensitivity and Anxiety‐Like Behavior

**Figure 7:**
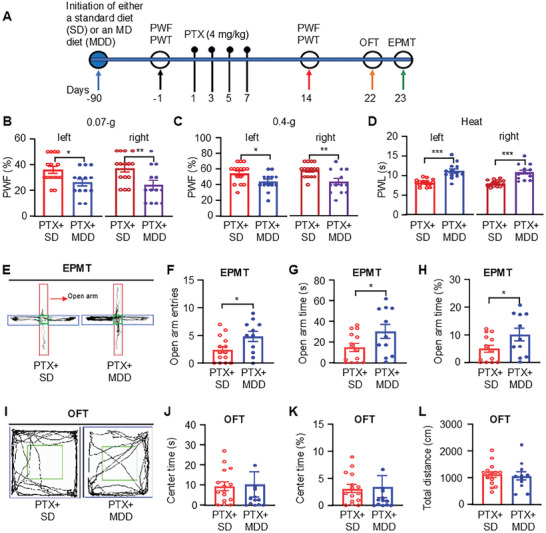
Supplementary methyl donor diets reduce paclitaxel (PTX)‐induced pain hypersensitivity and anxiety‐like behaviors in mice. A) Timeline of diet administration, drug treatments, and behavioral testing. B–D) Paw withdrawal responses to the 0.07 g von Frey filament (B), 0.4 g von Frey filament (C), and heat stimulation (D) in PTX‐treated mice after 3 months on a standard diet (SD) or a methyl donor diet (MDD). *n* = 11–15. *p < 0.05, **p < 0.01, ***p < 0.001, versus the PTX‐treated with normal diets group (PTX + normal), using two‐tailed unpaired *t*‐tests. E‐H) Schematic traces of the elevated plus‐maze test (EPMT) (E) and the number of entries into the open arms (F, the time (G) and the percentage of time (H) spent in the open arms in the EPMT. I–L) Schematic traces of the open field test (OFT) (I) and the time spent in the center (J) and the percentage of time spent in the center (K), and the total distance traveled in the center (L) in the OFT. *n* = 11–15. *p < 0.05, versus the PTX + normal diet group, using two‐tailed unpaired *t*‐tests.

Previous clinical and preclinical studies have consistently highlighted the therapeutic potential of S‐adenosine in alleviating inflammatory and neuropathic pain, as well as managing depression and other psychiatric disorders.^[^ ^21^ ^]^ Building on these findings, we investigated the efficacy of a methyl donor‐enriched diet in preventing PTX‐induced pain hypersensitivity and anxiety. Mice were provided with either a standard diet or a methyl donor‐rich diet for 3 months prior to PTX treatment (**Figure** **7**A). Notably, sustained consumption of the methyl donor diet significantly reduced mechanical allodynia and heat hyperalgesia in PTX‐treated mice (Figure 7B–D). Additionally, PTX‐treated mice on the methyl donor‐rich diet exhibited reduced anxiety‐like behaviors in the EPMT (Figure 7E–H). However, the methyl donor diet did not significantly alter the percentage of time spent in the center or the total travel distance in the OFT (Figure 7I–L). These findings suggest that a long‐term methyl donor‐enriched diet could be an effective strategy for alleviating PTX‐induced neuropathic pain and anxiety.

## 3 Discussion

**Figure 8:**
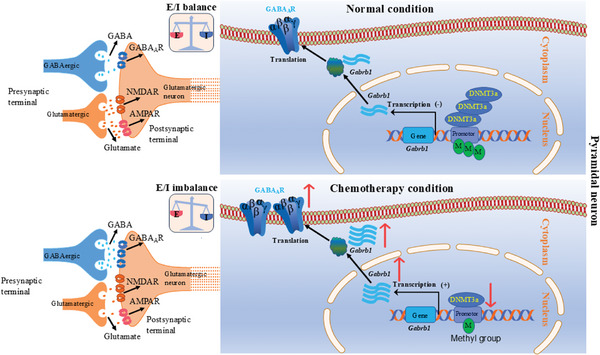
Proposed mechanism of DNMT3a involvement in pain and anxiety. In normal mPFC pyramidal neurons, DNMT3a‐dependent DNA methylation regulates GABA_A_ receptor expression. During chemotherapy, PTX‐induced downregulation of DNMT3a in the mPFC elevates *Gabrb1* mRNA levels, leading to increased GABA_A_ receptor expression in pyramidal neurons. This upregulation results in hyperactive inhibitory neurotransmission, disrupting the excitatory (E)/inhibitory (I) balance in the mPFC, thereby contributing to pain hypersensitivity and anxiety‐like behavior in PTX‐treated mice.

Pain and negative emotions are common and distressing symptoms experienced by cancer patients undergoing chemotherapy.^[^ ^2a,d^ ^]^ Understanding the underlying brain mechanisms could open new pathways for managing chemotherapy‐induced neuropathic pain and associated negative emotions. In this study, using a preclinical mouse model of PTX‐induced chemotherapy, we identified that PTX‐induced DNA hypomethylation and reduced DNMT3a expression led to the upregulation of GABA_A_Rβ1 and other GABA_A_ receptors. This upregulation resulted in hyperactive inhibitory neurotransmission within pyramidal neurons of the mPFC, ultimately contributing to pain hypersensitivity and anxiety‐like behavior in PTX‐treated mice (**Figure** **8**). Importantly, restoring DNMT3a expression in the mPFC alleviated PTX‐induced pain hypersensitivity and anxiety‐like behavior. Additionally, a long‐term methyl donor diet also provided relief from PTX‐induced pain and anxiety in mice. Considering the involvement of mPFC GABA_A_ receptors in neuropathic pain and negative emotions,^[^ ^20^ ^]^ this study provides insight into the mechanisms underlying dysregulation of GABA_A_R subunit expression and function during chemotherapy, suggesting a potential therapeutic target for managing chemotherapy‐induced pain and anxiety in clinical settings. It is notable, however, that PTX did not alter DNMT3a expression in female mice, indicating that this DNMT3a‐mediated epigenetic mechanism of PTX‐induced mPFC dysfunction may be gender‐specific, applying only to male mice.

PTX, a taxane‐class chemotherapeutic agent, is widely used for its potent anti‐tumor effects in treating various cancers, including breast, ovarian, and lung cancers.^[^ ^22^ ^]^ PTX interacts with microtubules in the cytoskeleton to enhance tubulin polymerization, leading to cancer cell apoptosis.^[^ ^22a^ ^]^ However, like many chemotherapeutic drugs, PTX induces not only peripheral neuropathy^[^ ^22b^ ^]^ but also central nervous system (CNS) toxicity, manifesting as chemotherapy‐induced cognitive impairment or “chemobrain”.^[^ ^2c^ ^]^ In the peripheral nervous system, PTX disrupts sensory neuron axonal microtubules, triggering a cascade of changes such as deregulated intracellular calcium, impaired axonal transport, mitochondrial dysfunction, altered neuropeptide secretion, and peripheral inflammation, all contributing to peripheral neuropathy and neuropathic pain.^[^ ^22b^ ^]^ Our findings demonstrate that CNS toxicity, specifically in the mPFC, is linked to altered pain perception and negative emotional states. Given previous evidence linking reduced mPFC activity to memory deficits and negative emotions in various pathological contexts,^[^ ^23^ ^]^ it is plausible that PTX‐induced cortical dysfunction contributes to multiple neural impairments in chemotherapy patients. Although no Fos expression changes were observed in the PAG after PTX treatment, resting‐state functional connectivity MRI studies have shown reorganization between the PAG and brainstem connections.^[^ ^24^ ^]^ Alternative methods may reveal changes in neuronal or synaptic activity within the PAG or other brain areas, providing further insights into PTX's central effects.

DNA methylation, a key epigenetic mechanism regulating gene expression, is catalyzed by DNMTs, enzymes that convert cytosine to 5‐mC.^[^ ^3^ ^]^ In cancer biology, research has highlighted the central role of epigenetic dysregulation in carcinogenesis, positioning epigenetic modifications as promising therapeutic targets.^[^ ^5a^ ^]^ While DNMT inhibitors have been explored as potential anticancer treatments,^[^ ^5a^ ^]^ our study reveals an alternative perspective: chemotherapy‐induced DNA hypomethylation and the downregulation of DNMT3a expression. Restoring DNMT3a levels, either through overexpression in the mPFC or a long‐term methyl donor‐rich diet, alleviated PTX‐induced pain hypersensitivity and anxiety‐like behavior. This suggests a therapeutic approach that diverges from conventional anticancer strategies. Moreover, numerous studies have documented sex‐specific DNA methylation patterns across contexts such as brain development, neurodegeneration, chemical exposure, and cancer.^[^ ^25^ ^]^ Observing sex‐biased DNMT3a expression in the mPFC following PTX treatment underscores the importance of considering sex differences in epigenetic modifications and their implications for therapeutic strategies.

In the human brain, DNA methylation is crucial for a range of functions, including synaptic plasticity, neuronal regeneration, learning, and memory.^[^ ^26^ ^]^ Importantly, DNA methylation has also been associated with pain‐related neuroplasticity.^[^ ^27^ ^]^ For instance, increased DNA methylation resulting from peripheral nerve injury can contribute to the development of neuropathic pain by silencing essential genes, such as the potassium channel gene Kcna2 and the opioid receptor gene *Oprm1*, in primary sensory neurons.^[^ ^6^, ^28^ ^]^ Previous studies on DNA methylation in the human PFC have demonstrated elevated methylation levels in genes related to synaptic transmission within neuronal nuclei.^[^ ^29^ ^]^ Moreover, peripheral nerve injury has been shown to induce global hypomethylation in the mPFC 6 months post‐injury,^[^ ^27^ ^]^ indicating that the neuroplastic changes associated with chronic pain from peripheral neuropathy may take time to manifest as DNA hypomethylation in the mPFC. In contrast, our findings indicate that global DNA hypomethylation and a reduction in DNMT3a levels in the mouse mPFC occur as early as 7 days after PTX treatment. This rapid onset of DNA hypomethylation shortly after PTX administration may not be a direct result of PTX‐induced peripheral neuropathy. Although PTX does not cross the blood‐brain barrier (BBB), it could stimulate an increased release of cytokines that readily penetrate the BBB and induce rapid structural and functional changes in the brain.^[^ ^30^ ^]^ While prior research has highlighted the significant roles of DNMT3a and DNMT1 in regulating anxiety‐like behavior in the mPFC,^[^ ^17^, ^31^ ^]^ our study emphasizes the specific contributions of DNA hypomethylation and reduced DNMT3a expression in pyramidal neurons to PTX‐induced pain hypersensitivity and anxiety. However, it seems that DNMT3a and DNA hypomethylation in the mPFC are not involved in the development of PTX‐induced depression. A previous study showed that optogenetic activation of glutamatergic neurons in the mPFC does not affect depression‐like behavior in mice during the FST, while selective activation of mPFC projections to the brainstem DRN elicits antidepressant effects observed in the FST.^[^ ^32^ ^]^ Furthermore, acetylcholine and cholinergic signaling in the mPFC have been clinically associated with depression.^[^ ^33^ ^]^ Thus, other molecular targets may play a role in the onset of PTX‐induced depression, potentially in a cell‐type or circuit‐specific manner.

The mPFC plays a crucial role in modulating pain, emotion, and cognition.^[^ ^9^, ^10^ ^]^ Previous research has highlighted the crucial role of excitatory and inhibitory synaptic changes in the mPFC in pain modulation.^[^ ^34^ ^]^ In this experiment, we also preliminarily explored the significance of synaptic changes in chemotherapy‐induced pain, confirming that PTX‐induced chemotherapy pain reduced the E/I ratio in the mPFC, which could be reversed by DMNT3a, concurrently exhibiting an analgesic effect behaviorally, thereby linking synaptic changes to behavioral phenotypes. Specifically, the glutamate pathway in the mPFC exhibits analgesic properties mediated by the GABA_A_R.^[^ ^35^ ^]^ Mammalian systems contain nineteen distinct GABA_A_R subunits, including six α, three β, three γ, along with δ, ε, θ, π, and three ρ subunits, which combine to form various GABA_A_R subtypes. Among these, the most prevalent subtypes consist of α1, β2, and γ2 subunits, while those containing α2, β1, and γ1 subunits have also been identified.^[^ ^36^ ^]^ The current study demonstrates that PTX induces the upregulation of multiple GABA_A_R subunits, including α1‐6, β1‐2, and γ1‐2 in the mPFC, a change that can be reversed by DNMT3a overexpression. As one of the most extensively researched pharmacological targets, GABA_A_ receptors offer a diverse array of therapeutic agents, including agonists, antagonists, and modulators, refined for treating neuropsychiatric disorders such as anxiety and depression.^[^ ^37^ ^]^ Numerous pharmacological studies and clinical data have confirmed the antinociceptive and anxiolytic properties of GABA_A_R antagonists.^[^ ^20^, ^38^ ^]^ Our findings support their potential use in treating chemotherapy‐related neuropathic pain and anxiety. Given the role of GABA_A_ receptors in pain modulation, our study highlights the therapeutic potential of DNA methylation‐mediated epigenetic regulation in restoring excitatory/inhibitory balance in the mPFC, thereby modulating pain sensation and pain‐related anxiety (Figure 8). Specifically, we demonstrated that a long‐term (3‐month) methyl donor diet effectively alleviates PTX‐induced neuropathic pain and anxiety‐like behaviors in mice. Other studies also support the positive impact of methyl‐donor diets in managing pain, emotional disorders, and cognitive impairments in both humans and animals.^[^ ^21^, ^39^ ^]^ However, when we assessed pain and anxiety‐like behavior 1 month after continuous feeding with the methyl donor diet, we did not observe a significant effect on PTX‐induced neuropathic pain and anxiety (data not shown). We hypothesize that long‐term consumption of methyl donors helps maintain a higher baseline level of DNA methylation, thereby counteracting the gradual DNA hypomethylation that occurs following PTX treatment.

In summary, our study uncovers a DNMT3a‐mediated epigenetic mechanism that drives the upregulation of GABA_A_R and hyperactivated inhibitory synaptic transmission in pyramidal neurons of the mPFC, contributing to PTX‐induced pain hypersensitivity and anxiety‐like behavior in mice. As a result, DNMT3a and GABA_A_R emerge as promising targets for managing neuropathic pain and anxiety during chemotherapy. In recent years, DNMT inhibitors have been developed for cancer treatment.^[^ ^5^ ^]^ Our findings underscore the necessity for developing DNMT agonists or therapeutic strategies aimed at increasing DNA methylation to treat neurological disorders. Moreover, the distinct roles of various GABA_A_R subunits warrant further investigation for the development of specific GABA_A_R antagonists or modulators.

## 4 Experimental Section

### Materials

PTX, oxaliplatin (OXA), and bicuculline were purchased from MedChemExpress (Shanghai, China). Cremophor EL, complete Freund's adjuvant (CFA), and picrotoxin were sourced from Sigma–Aldrich (St. Louis, MO, USA). 6‐cyano‐7‐nitroquinoxaline‐2,3‐dione (CNQX) was sourced from Tocris Cookson (Bristol, UK). Cell culture‐medium was sourced from Gibco/Thermo Fisher Scientific (Waltham, MA, USA). Fetal bovine serum, B‐27 supplement, penicillin, streptomycin, L‐Glutamine, and poly‐D‐lysine were sourced from Beyotime Biotechnology (Shanghai, China). All virus strains were acquired from BrainVTA (Wuhan, China). The antibodies were purchased from various companies, as detailed in Table S1 (Supporting Information). All primers were synthesized by AuGCT (Peking, China), and their sequences are listed in Table S2 (Supporting Information).

### Animals

Adult C57BL/6J wild‐type male mice (8–10 weeks old; Laboratory Animal Center of Xi'an Jiaotong University, Xi'an, Shaanxi, China), CaMKII‐Cre (B6J.Cg‐Tg(Camk2a‐cre)T29‐1Stl/J; RRID: IMSR_JAX:005359, The Jackson Laboratory, Bar Harbor, ME, USA) and GAD‐Cre (B6J.Cg‐*Gad2tm2(cre)Zjh*/J; RRID: IMSR_JAX:028867, The Jackson Laboratory) were used in the study. All mice were housed in a controlled environment with a 12‐h light‐dark cycle, maintained at an optimal temperature of 25 ± 1 °C, and provided with adequate ventilation. The animals had free access to food and water throughout the study. All experimental procedures were approved by the Institutional Animal Ethics Committee of Xi'an Jiaotong University Health Science Center (no. 2018–334) and strictly adhered to the ethical standards set by the International Association for the Study of Pain. Every effort was made to minimize the number of animals used and to reduce any potential suffering.

### Chemotherapy‐Induced Neuropathic Pain Models

For PTX treatment, a stock solution of PTX (MedChemExpress) at 6 mg mL^−1^ was prepared using a 1:1 ratio of Cremophor EL (Sigma–Aldrich) and ethanol, in accordance with a previously established protocol.^[^ ^8^ ^]^ Immediately before administration, this PTX concentrate was diluted to a final concentration of 0.4 mg mL^−1^ with 0.9% sterile sodium chloride (NaCl). PTX (4 mg kg^−1^) was then administered intraperitoneally (i.p.) every other day for a total of 4 injections. For control mice, a vehicle solution composed of Cremophor EL and ethanol in a 1:1 ratio, further diluted with 0.9% sterile NaCl to a final concentration of 33.3%, was used.

Oxaliplatin (OXA, MedChemExpress) was prepared following a previously established protocol.^[^ ^40^ ^]^ Initially, OXA was dissolved in a 5% glucose solution to create a stock solution with a concentration of 3 mg mL^−1^. This stock solution was stored at −20 °C for up to 14 days. Before administration, the stock solution was further diluted with a 5% glucose solution to achieve a final concentration of 0.6 mg mL^−1^. A dose of 6 mg kg^−1^ of OXA was then administered intraperitoneally on alternate days for a total of 4 consecutive injections. For the control group, a 5% glucose solution was used as the vehicle.

### Spinal Nerve Ligation (SNL)‐Induced Neuropathic Pain Model

The SNL surgery was performed as described in the previous study.^[^ ^41^ ^]^ Briefly, mice were anesthetized using a 2.5% isoflurane and oxygen mixture, after which the L4 transverse process on the left side of the spine was excised. The L4 spinal nerve was then ligated with a 7‐0 silk thread and transected at its distal end. The sham surgery group underwent the same surgical protocol, but without the ligation and transection of the nerve.

### CFA‐Induced Inflammatory Pain Model

The CFA‐induced inflammatory pain model in mice was established by injecting an oil‐saline emulsion (1:1) containing CFA (10 µL/paw; Mycobacterium tuberculosis; Sigma–Aldrich) into the plantar surface of the left hind paw, following previously described methods.^[^ ^42^ ^]^ For the control group, 10 µL of 0.9% sterile NaCl solution was administered instead.

### Virus Information and Microinjection

Following anesthesia, the mice were placed in a stereotaxic apparatus, and the skin was incised to expose the Bregma (anterior fontanelle) on the skull surface. Using the mouse brain stereotaxic atlas, the mPFC was precisely located at the following coordinates: anterior‐posterior (AP) = +2.0 mm, medial‐lateral (ML) = ±0.4 mm, and dorsal‐ventral (DV) = −2.3 mm from the Bregma. Bilateral drill holes were made at these sites, and a glass electrode connected to a Hamilton microsyringe (Shanghai Gaoge, China) was gently inserted into the target region. A total volume of 0.25 µL of AAV vectors (per side) was administered at a controlled rate of 0.025 µL min^−1^ using a microsyringe pump (ZSDichuang, Peking, China). After a 10‐min injection period, the needle was left in place for an additional 10 min to ensure even distribution of the solution. The needle was then slowly withdrawn, and the incision was sutured. Following the injection, the mice were housed for three weeks to allow for adequate viral transfection, preparing them for subsequent experimental procedures. Detailed information about the viral vectors used is provided in Table S1 (Supporting Information).

### Cannula Implantation and Local Drug Administration in mPFC

After anesthetizing and positioning the mice, a double‐guide cannula (Catalog No. 62523; RWD Life Science, Shenzhen, China) was stereotaxically implanted 0.5 mm above the mPFC using the coordinates: AP = +2.0 mm, ML = ±0.4 mm, and DV = −1.8 mm from the Bregma. A dummy cannula was then inserted into the guide cannula. The animals were returned to the recovery area for a minimum of 7 days to allow for post‐surgical healing. For local drug administration, a bilateral injection cannula attached to a 1 µL microsyringe was inserted into the guide cannula, extending 0.5 mm beyond its tip. A ZSDichuang infusion pump was used to bilaterally administer a solution containing either bicuculline (100 ng, MedChemExpress), siRNA (500 ng), or a control vehicle into the mPFC at a volume of 0.5 µL per side. After infusion, the injector was kept in place for an additional 5 min to prevent backflow. The siRNAs were specifically designed and synthesized by Genepharma (Shanghai, China), with the following sequences: *Dnmt3a* siRNA (5'‐AGAUGUUCUUUGCCAAUAATT‐3', 5'‐UUAUUGGCAAAGAACAUCUGG‐3') and a negative control (NC) siRNA (5'‐UUCUCCGAACGUGUCACGUTT‐3', 5'‐ACGUGACACGUUCGGAGAATT‐3'). For siRNA delivery, Entranster‐in vivo transfection reagent (Cat#18668‐11‐1, Engreen Bio‐system, Beijing, China) was used, with a composition ratio of *Dnmt3a* siRNA: 20% glucose: transfection reagent at 2:1:1, resulting in a final concentration of 500 ng µL^−1^.

### Methyl Donor Diet and Breeding Protocol

High‐methyl donor chows, obtained from Professionals for Lab Animal Diets (Medicine, Yangzhou, China), were enriched with methionine, zinc carbonate, folic acid, betaine, cyanocobalamin, choline, and genistein, as previously described.^[^ ^39b^ ^]^ Mice consuming either the high‐methyl donor diet or the standard diet began their respective dietary regimens 3 months prior to PTX treatment and continued throughout the entire experimental period.

### Behavioral Tests

Mechanical stimulus‐induced pain hypersensitivity was assessed using two calibrated von Frey filaments (0.07 and 0.40‐g; Aesthesio, San Jose, CA, USA), following a previously established methodology.^[^ ^28^ ^]^ Mice were placed in a Plexiglas chamber with an elevated mesh screen for 30 min. Each hind paw was stimulated ten times with each von Frey filament separately. The paw withdrawal frequency (PWF) was calculated by determining the percentage of positive withdrawal responses out of the ten trials, using the formula: ([number of paw withdrawals/10 trials] × 100%).

Heat‐induced pain hypersensitivity was evaluated using the Hargreaves test. Mice were placed on a glass plate inside a Plexiglas chamber for 30–40 min. A radiant heat source from a Model 336 Analgesia Meter (IITC Inc./Life Science Instruments, Woodland Hills, CA, USA) was then applied to the hind paw. The paw withdrawal latency (PWL) was recorded as the time elapsed between the onset of the light beam and the lifting of the paw. Each hind paw was tested over five trials, with a 5‐min interval between trials. To prevent tissue injury, the radiant heat was automatically shut off after 20 s.

Cold‐induced pain hypersensitivity was assessed using a cold plate test, as previously described.^[^ ^28^ ^]^ Mice were placed on an ice‐cold aluminum plate (Zheng‐Hua Biologic, Huaibei, China) maintained at 0°C. The latency from the moment the hind paw was placed on the cold plate until a positive response—such as flinching, paw shaking, or jumping—was recorded. Each hind paw underwent three trials, with a 10‐min interval between trials. A cut‐off time of 20 s was set to prevent potential tissue injury.

Locomotor function was evaluated by observing placing, grasping, and righting reflexes, as previously reported.^[^ ^43^ ^]^ In the placing test, the mouse's ability to promptly place its hind paws on a table when positioned with its hind limbs hanging over the edge was assessed. The grasping test involved determining whether the mouse could grasp a wire when placed on a wire grid. The righting test examined the mouse's ability to restore its righting reflex (flipping to a prone position) when initially placed on its back (supine position) in an unrestrained manner. Each reflex was tested five times, with each successful attempt receiving a score of one.

The rotarod test, performed using an accelerating rotarod instrument (Panlab/Harvard Apparatus, Holliston, MA, USA), was utilized to assess motor coordination and balance, as previously described.^[^ ^44^ ^]^ After 3 consecutive days of training, the mice underwent formal testing on day 14 post‐vehicle or PTX treatment. During the training sessions, the mice were placed on rotating rods (3 cm in diameter) and trained for 15 min at a constant speed of 15 revolutions per minute (rpm). On the test day, the rods accelerated from 4 to 40 rpm over a 5‐min period. Each mouse completed three trials, with a 15‐min rest interval between trials. The rotational speeds and retention times at which each mouse fell off the rod during the trials were recorded and averaged.

The open field test (OFT) was conducted with mice as previously described.^[^ ^20^ ^]^ Mice were placed in the center of a cubic chamber measuring 50 cm × 50 cm × 40 cm and allowed to explore freely for 5 min. Their activity was recorded and tracked using Smart 3 video tracking software (Panlab/Harvard Apparatus). The time spent and the percentage of time spent in the central area (a 25 cm × 25 cm region at the center of the chamber) were measured to assess anxiety‐like behavior, while the total distance traveled was recorded as an indicator of locomotion.

The elevated plus maze test (EPMT) was conducted according to established protocols to assess anxiety‐like behavior in mice.^[^ ^8^, ^20^ ^]^ The EPM apparatus consisted of a central square (8 cm × 8 cm), two open arms (30 cm long × 8 cm wide), and two closed arms (30 cm long × 8 cm wide), all made from black acrylic. The plus‐shaped platform was elevated 50 cm above the ground. During the test, mice were individually placed in the center of one of the open arms and allowed to explore freely for 5 min. The number of entries into the open arms, as well as the time spent and the percentage of time spent in the open arms, were recorded and analyzed using an automated system paired with Smart 3 video tracking software (Panlab/Harvard Apparatus).

The forced swim test (FST) was conducted according to previously described protocols to assess depression‐like behavior in mice.^[^ ^8^ ^]^ Mice were gently placed into individual glass cylinders, each measuring 21 cm in height, with an inner diameter of 14 cm, an outer diameter of 15 cm, and a thickness of 0.5 cm. They were allowed to swim for 6 min in the cylinders filled with water to two‐thirds of their capacity, maintained at a temperature of 24 ± 1 °C, to facilitate acclimatization to the environment. The duration of immobility during the last 4 min of the test was recorded.

### Primary Cortex Neuron Culture

Three‐ to four‐week‐old mice were euthanized after anesthesia with 2.5% isoflurane. The cerebral cortex from two mice was excised and placed in cold Hanks’ Balanced Salt Solution (HBSS) (Gibco/Thermo Fisher Scientific). The tissue was digested using a 0.25% trypsin solution free of ethylenediaminetetraacetic acid (Beyotime Biotechnology). Following trituration and centrifugation, the dissociated cells were cultured in Neurobasal A medium (Gibco/Thermo Fisher Scientific), supplemented with 10% fetal bovine serum, B‐27 supplement (Gibco/Thermo Fisher Scientific), 100 units/mL penicillin, 100 mg/mL streptomycin, and 200 mM L‐glutamine (Beyotime Biotechnology). This mixed Neurobasal A medium was then plated onto six‐well plates that had been pre‐treated with 100 mg/mL poly‐D‐lysine (Beyotime Biotechnology). The cells were incubated overnight in an incubator maintained at 37 °C with 95% O₂ and 5% CO₂. After dilution of the virus (2 µL in 200 µL), AAV‐EGFP or AAV‐*Dnmt3a* was added to 1 mL of medium in the wells of the six‐well plate. After 2 days, the cultured cells adhering to the bottom of the six‐well plate were collected.

### Methylated 5‐mC DNA Quantification

The mice were euthanized after anesthesia with 2.5% isoflurane, and mPFC tissues were carefully dissected and lysed using a buffer containing protease K (Epigentek Group, Farmingdale, NY, USA). The precipitated DNA was isolated by treating the samples with isopropyl alcohol, washed with 70% ethanol, and then dissolved in deionized water. According to the instructions of the MethylFlash Methylated 5‐mC DNA Quantification Kit (Colorimetric, Epigentek Group), 100 ng of sample DNA, along with positive and negative controls, were added to designated wells using the binding solution and incubated at 37 °C for 90 min. The methylated DNA was captured by adding capture antibody, detection antibody, and enhancer solution. Afterward, all samples were incubated with the developer solution in a light‐shielded environment at room temperature for 1 to 10 min, and the enzyme reaction was halted using the stop solution. Absorbance was measured using a Multiskan FC microplate reader (Thermo Fisher Scientific). The relative methylation level of the DNA sample was determined by calculating the percentage of 5‐mC in the total DNA.

### Western Blotting

The mice were euthanized after anesthesia with 2.5% isoflurane, and mPFC tissues were collected and stored at −20 °C in AllProtect Nucleic Acid and Protein Stabilization Reagent for Animal Tissue (Beyotime Biotechnology). The tissues were homogenized in a cold tissue homogenizer (Shanghai Jingxin, Shanghai, China) using radioimmunoprecipitation assay (RIPA) lysis buffer supplemented with a protease inhibitor cocktail (MedChemExpress) and phenylmethylsulfonyl fluoride (PMSF) from Beyotime Biotechnology. The crude homogenate was centrifuged at 1000 g for 15 min, yielding a supernatant containing cytoplasmic proteins and a precipitate containing cytonuclear proteins. A Bicinchoninic Acid Assay (BCA) Protein Assay Kit (Bioss, Peking, China) was used to quantify the protein concentration. Following denaturation at 99 °C for 5 min, the proteins were loaded onto a glycine‐SDS‐PAGE gel (Beyotime Biotechnology) and subsequently transferred to a 0.45‐µm nitrocellulose membrane (Upstate/EMD Millipore, Darmstadt, Germany). The membrane was then blocked with 5% skim milk for 2 h. Depending on specific experimental requirements, the membrane was incubated overnight with the respective primary antibodies, followed by horseradish peroxidase (HRP)‐conjugated anti‐rabbit (1:5000, Sigma–Aldrich) and anti‐mouse (1:5000, DIYIBio, Shanghai, China) secondary antibodies for 2 h. Finally, the blots were visualized using an Enhanced Chemiluminescence (ECL) Ultra kit (Upstate/EMD Millipore) and captured with the Champchemi System and SageCapture software (SageCapture Service for Life Science, Beijing, China). Image J software (Wayne Rasband, National Institutes of Health, Bethesda, MD, USA) was utilized for blot analysis. Histone H3 and glyceraldehyde‐3‐phosphate dehydrogenase (GAPDH) served as internal controls for nuclear and cytoplasmic proteins, respectively, to normalize the target blot. The average value in the control group was set to 100% for further comparisons.

### Real‐Time Quantitative Polymerase Chain Reaction (RT‐qPCR)

The mice were euthanized after anesthesia with 2.5% isoflurane, and mPFC tissues were promptly dissected and preserved in RNAlater (Beyotime Biotechnology). Total RNA was extracted from the mPFC tissues using an RNAeasy RNA extraction kit (Beyotime Biotechnology) and quantified with a Nanodrop spectrophotometer (Thermo Fisher Scientific). Subsequently, 500 ng of total RNA was reverse transcribed into cDNA using oligo(dT) primers and the RevertAid First Strand cDNA Synthesis Kit (Thermo Fisher Scientific). A 20 µL reaction mixture was prepared for real‐time PCR amplification in a BIO‐RAD CFX96 system (Bio‐Rad, Hercules, CA, USA), containing 20 ng of cDNA, 250 nM of forward and reverse primers, and 10 µL of BeyoFast SYBR Green qPCR Mix (Beyotime Biotechnology). The amplification protocol included an initial denaturation step at 95 °C for 3 min, followed by 40 cycles of denaturation at 95 °C for 10 s, annealing at 60 °C for 30 s, and extension at 72 °C for 30 s. To ensure accurate data interpretation, all results were normalized to Gapdh, which served as the internal control. The mRNA levels in the control group were averaged, and the relative expression levels in each experimental group were calculated using the ΔCt method (2^−ΔCt^), comparing them to the average mRNA levels of the control group.

### Chromatin Immunoprecipitation (ChIP) Assay

The mice were euthanized following anesthesia with 2.5% isoflurane, and the mPFC tissues were promptly dissected and quickly frozen in liquid nitrogen. Bilateral mPFC tissues from three mice were pooled and homogenized in 0.01 M phosphate‐buffered saline (PBS) supplemented with a protease inhibitor cocktail II. ChIP assays were conducted using the EZ ChIP Kit (Upstate/EMD Millipore) according to the manufacturer's guidelines. To stabilize the homogenate, 1% formaldehyde was added for crosslinking, which was subsequently quenched by adding a 0.125 M glycine solution. Following centrifugation, the resulting pellet was lysed with SDS lysis buffer containing the protease inhibitor cocktail and sonicated until the DNA fragments reached an average length of 200 to 1000 bp. Prior to immunoprecipitation, samples were pre‐cleared with resuspended protein A/G agarose beads. Immunoprecipitation was performed overnight at 4 °C using either 5 µg of rabbit antibodies specific to DNMT3a (1:1000, Cell Signaling Technology, Danvers, MA) or 5 µg of normal mouse IgG as a negative control. An Input group, comprising 10% of the total sample, served as a positive control. Subsequently, purified DNA fragments were analyzed using PCR or real‐time PCR with custom‐designed primers. The relative mRNA levels in each group were calculated using the ΔCt method (2^−ΔCt^), compared against the average mRNA levels in the control group. Independent ChIP experiments were replicated using mPFC tissues from three mice per group.

### Bisulfite Sequencing

The mice were euthanized following anesthesia with isoflurane, and the bilateral mPFC tissues from mice were collected for DNA extraction and sent to BGI Genomics Inc. (Wuhan, China) for bisulfite sequencing experiments. Tissues from three mice were pooled to serve as biological replicates. Genomic DNA was isolated using the TIANamp Genomic DNA Kit (TIANGEN, Peking, China) according to the manufacturer's instructions. This DNA was then converted into bisulfite‐modified DNA using the EpiTect Plus DNA Bisulfite Kit (QIAGEN, Holland, Germany), which is specifically designed for methylation transformation. The target region of the *Gabrb1* gene promoter and the 5’‐untranslated region (5’‐UTR), spanning from ‐959 bp to ‐856 bp and encompassing five CpG sites, was subsequently amplified. For pyrosequencing, a unique primer pair for Gabrb1, modified with 5’‐biotin, was employed to amplify the bisulfite‐converted DNA. Amplification was performed in an ABI 9700 PCR System (Thermo Fisher Scientific) using a 50 µL reaction mixture that included 2 µL of bisulfite‐converted DNA, 1 µL of PCR primers, 2 µL of dNTP mix, 10 µL of 5× PCR GC buffer, and 0.2 µL of 5 U Taq DNA polymerase (KAPA Biosystems, Boston, CA, USA). The optimized PCR cycling conditions were as follows: initial denaturation at 95 °C for 3 min, followed by 40 cycles of denaturation at 95 °C for 30 s, annealing at 50 °C for 30 s, and extension at 72 °C for 1 min. The resulting pyrosequences were analyzed using the PyroMark Q48 Autoprep sequencing software (QIAGEN) to accurately determine the methylation percentage at each CpG site within the targeted region.

### Immunofluorescence

Mice were transcardially perfused with 4% paraformaldehyde after anesthesia with isoflurane. To induce acute Fos expression in the spinal cord, a size #1 paintbrush was used to brush the left paw for 1 min, 2 h before perfusion. The intact brains and/or lumbar spinal cords were then dissected for subsequent post‐fixation and dehydration. Cryosections of 20 µm thickness were produced using a Leica CM1860 cryostat (Leica, Wetzlar, Germany). Brain sections containing the mPFC region were directly mounted onto gelatin‐coated slides. For single‐labeling of calcium/calmodulin‐dependent protein kinase II alpha (CaMKIIα), gamma‐aminobutyric acid (GABA), or c‐Fos, the sections were blocked with 10% normal goat serum for 1 h. They were then incubated overnight at 4 °C with the respective primary antibodies: rabbit anti‐CaMKIIα (1:100, Immunoway, Plano, TX, USA), rabbit anti‐GABA (1:500, Sigma–Aldrich), or rabbit anti‐c‐Fos (1:400, Abcam, Cambridge, MA, USA). Afterward, the sections were incubated with secondary antibodies conjugated to Cy3 (1:1000, Upstate/EMD Millipore) or Alexa Fluor 488 (1:200, Abcam) for 2 h at room temperature. For double‐labeling of GABA_A_Rβ1 with excitatory amino acid carrier 1 (EAAC1), mPFC sections were incubated overnight at 4 °C with mouse anti‐GABA_A_Rβ1 (1:100, Abcam) and goat anti‐EAAC1 (1:1000, Upstate/EMD Millipore) antibodies. This was followed by incubation with a mixture of secondary antibodies: goat anti‐mouse conjugated to Alexa Fluor 488 (1:200, Abcam) and rabbit anti‐goat conjugated to Cy3 (1:1000, Upstate/EMD Millipore). Negative controls were included by omitting the primary antibodies. The slides were coverslipped using SouthernBiotech Fluoromount‐G (SouthernBiotech, Birmingham, AL, USA) or an antifade mounting medium containing 4',6‐diamidino‐2‐phenylindole (DAPI; Beyotime Biotechnology). Digital images were captured using an Axio Scope A1 fluorescence microscope (ZEISS, Carl Zeiss, Germany), equipped with X‐Cite 120Q Fluorescence Lamp Illuminators (Excelitas, Mississauga, Canada) and ZEN 2.3 lite software. The number of single‐ or double‐labeled cells was manually counted or quantified using ImageJ software, with images acquired from 4 to 6 sections per mouse.

### RNAscope and Immunofluorescence Co‐Detection

Following anesthesia with isoflurane, the mice were perfused with 4% paraformaldehyde. The brains were then dissected, post‐fixed, and dehydrated. Cryosections with a thickness of 15 µm were obtained using a Leica CM1860 cryostat. In situ hybridization was performed on the frozen tissue sections using the RNAscope Multiplex Fluorescent Detection Reagent Kit (ACD Biotech, Newark, CA, USA), following the manufacturer's instructions. Additionally, the Co‐detection Target Retrieval Reagent Kit (ACD Biotech) was employed for concurrent immunofluorescence detection. The frozen sections underwent further post‐fixation in 10% neutral formalin and dehydration through a series of graded ethanol concentrations (50%, 70%, and 100%). After pretreatment with hydrogen peroxide and target retrieval reagents, the Mm‐Dnmt3a probe (ACD Biotech) was applied for 2 h. For the concurrent detection of *Dnmt3a* mRNA and specific proteins, the sections were first incubated with primary antibodies, including mouse anti‐CaMKIIα (1:100, Cell Signaling Technology), rabbit anti‐GABA (1:500, Sigma–Aldrich), or mouse anti‐GABA_A_Rβ1 (1:100, Abcam), in Co‐detection Antibody Diluent (ACD Biotech). Following this incubation, the sections were exposed to protease and then treated with secondary antibodies: donkey anti‐mouse conjugated to FITC (1:400, Upstate/EMD Millipore) or donkey anti‐rabbit conjugated to Alexa Fluor 488 (1:200, Abcam). The sections were subsequently hybridized with the Mm‐Dnmt3a probe for 2 h, followed by consecutive treatments with AMP1, AMP2, and AMP3 for 15 to 30 min each. To fluorescently label the probe, the sections were incubated with horseradish peroxidase‐channel 1 (HRP‐C1) for 15 min, followed by incubation with Opal 570 (1:1500, Perkin Elmer, Waltham, MA, USA) for 30 min. All hybridization and incubation steps were conducted at 40°C in a hybridization oven, with moisture maintained using a wet box. The sections were then mounted and visualized under an Axioscope A1 fluorescence microscope (ZEISS). Images were captured using an X‐Cite 120Q Fluorescence Lamp Illuminator (Excelitas Technologies) coupled with ZEN 2.3 lite software. The acquired images were derived from 4 to 6 brain slices from three different mice.

### Brain Slice Preparation and In Vitro Whole‐Cell Patch‐Clamp Recording

Coronal brain slices (300 µm) containing the mPFC were prepared as described previously.^[^ ^45^ ^]^ Briefly, mice were anesthetized with 1–2% isoflurane within a short time and sacrificed by decapitation. The whole brain was rapidly removed and transferred into ice‐cold oxygenated (95% O_2_ and 5% CO_2_) cutting solution (in mM: 252 sucrose, 2.5 KCl, 6 MgSO_4_, 0.5 CaCl_2_, 25 NaHCO_3_, 1.2 NaH_2_PO_4_, and 10 glucose, pH 7.3 to 7.4) for a short time. For the making of coronal brain slices, the brain was trimmed and glued onto the ice‐cold platform of a vibrating tissue slicer (Leica VT1200S). Then 300 µm‐thickness slices containing the mPFC were cut and transferred to a submerged recovery chamber with oxygenated (95% O_2_ and 5% CO_2_) artificial cerebrospinal fluid (ACSF) (in mM: 124 NaCl, 2.5 KCl, 2 CaCl_2_, 2 MgSO_4_, 25 NaHCO_3_, 1 NaH_2_PO_4_, and 10 glucose, pH 7.3 to 7.4) at room temperature for at least 1 h incubation before conducting whole‐cell patch‐clamp recordings. The whole‐cell patch‐clamp recordings were performed as previously described.^[^ ^45^, ^46^ ^]^ Experiments were performed in a recording chamber placed in an Olympus BX51W1 microscope with infrared differential interference contrast (DIC) optics to visualize whole‐cell patch‐clamp recording. Evoked excitatory post‐synaptic currents (EPSCs) were recorded from layer V neurons with an Axon 200B amplifier (Molecular Devices, Union City, CA, USA), and the stimulations were delivered by a bipolar tungsten stimulating electrode placed in layer II/III of mPFC. Miniature EPSCs (mEPSCs) were recorded with the membrane potential holding at −70 mV, and bath presence of picrotoxin (100 µM) and tetrodotoxin (TTX, 1 µM). Miniature inhibitory post‐synaptic currents (mIPSCs) were recorded with the membrane potential holding at 0  mV, and bath presence of CNQX (20 µM) and TTX (1 µM). The recording pipettes (3–5 MΩ) were filled with a K^+^‐ internal solution containing (in mM) 145 K‐gluconate, 5 NaCl, 1 MgCl_2_, 0.2 EGTA, 10 HEPES, 2 Mg‐ATP, 0.1 Na_3_‐GTP (adjusted to pH 7.2 with KOH, 290 mOsmol) for recording mEPSCs. The recording pipettes were filled with Cs^+^‐internal solution (in mM) 112 Cs‐Gluconate, 5 TEA‐Cl, 3.7 NaCl, 0.2 EGTA, 10 HEPES, 2 Mg‐ATP, 0.1 Na_3_‐GTP, and 5 QX‐314 (adjusted to PH 7.2 with CsOH, 290 mOsmol) were used for recording mIPSCs. The evoked E‐I ratio was defined with the ratio of the amplitude of evoked EPSCs (eEPSCs) and evoked IPSCs (eIPSCs), when the neurons were held in the −70 mV for recording eEPSCs and held in the 0 mV for recording eIPSCs respectively, with the recording pipettes filled with Cs^+^‐internal solution and bath with normal ACSF. The GABA_A_R‐mediated eIPSCs were recorded with the bath presence of CNQX (20 µM) and APV (50 µM) with the membrane potential holding at 0 mV. Clampex version 10.3 and Clampfit version 10.2 software (Molecular Devices) were used to collect and analyze data. Data were discarded if access resistance changed 15% during an experiment. Data were filtered at 1 kHz, and digitized at 10 kHz using the digidata 1440A.

### Statistical Analysis

The processed data from all experiments were statistically analyzed using GraphPad Prism 9 software (GraphPad, San Diego, CA, USA). For behavioral tests, a minimum of six mice were included in each group, while for molecular studies, at least three mice were used per group. Comparisons between two groups were performed using a two‐tailed unpaired Student's *t*‐test. For comparisons involving more than two groups, either a one‐way analysis of variance (ANOVA) or a two‐way repeated measures (RM) ANOVA was employed. When ANOVA indicated significant differences, *post hoc* Tukey's test was applied to compare the mean values. Mice were randomly assigned to the various treatment groups. All data are presented as the mean ± standard error of the mean (SEM), with a P‐value of less than 0.05 considered statistically significant.

## Conflict of Interest

The authors declare no conflict of interest.

## Author Contributions

L.T. and X.‐H.L. contributed equally to this work. L.T. and X.‐H.L. wrote the draft. L.T. performed most of the experiments and analyzed the data. X.‐H.L. performed and analyzed electrophysiological data. Y.‐L.Z. maintained the mouse lines. H.‐Y.Y. assisted with the behavior tests and Western blotting experiments. X.‐R.L., R.Y., X.‐M.H., and X.Z., assisted with tissue harvesting and immunofluorescence experiments. F.‐Q.H. provided instructive advice and edited the manuscript. T.C. oversaw the electrophysiological data and edited the manuscript. L.L. conceived the study, designed and supervised the experiments, and substantially edited the manuscript. All authors read and approved the final manuscript.

## Supporting information



Supporting Information

## Data Availability

The data that support the findings of this study are available from the corresponding author upon reasonable request.
