# Supplementary material for: DNMT3a Downregulation Ttriggered Upregulation of GABAA Receptor in the mPFC Promotes Paclitaxel‐Induced Pain and Anxiety in Male Mice
Source: Adv Sci (Weinh). 2024 Dec 16;12(5):2407387. doi: 10.1002/advs.202407387 (PMC11791956; doi:10.1002/advs.202407387)
Supplement: Supplementary file 1 — Supporting Information [file ADVS-12-2407387-s001.pdf]

ADVANCED SCIENCE
Open Access

Supporting Information

for *Adv. Sci.*, DOI 10.1002/advs.202407387

DNMT3a Downregulation Ttriggered Upregulation of $GABA_A$ Receptor in the mPFC Promotes Paclitaxel-Induced Pain and Anxiety in Male Mice

Lixia Tian, Xu-Hui Li, Yu-Long Zhao, Hui-Yuan Yi, Xue-Ru Liu, Rongrong Yao, Xue-Mei Hou, Xuan Zhu, Fu-Quan Huo, Tao Chen* and Lingli Liang**

Supporting Information

for Adv. Sci.,

DNMT3a downregulation-triggered Upregulation of $GABA_A$ Receptor in the mPFC Promotes Paclitaxel-induced Pain and Anxiety in Male Mice

Lixia Tian, Xu-Hui Li, Yu-Long Zhao, Hui-Yuan Yi, Xue-Ru Liu, Rongrong Yao, Xue-Mei Hou, Xuan Zhu, Fu-Quan Huo, Tao Chen*, Lingli Liang**

Figure S1

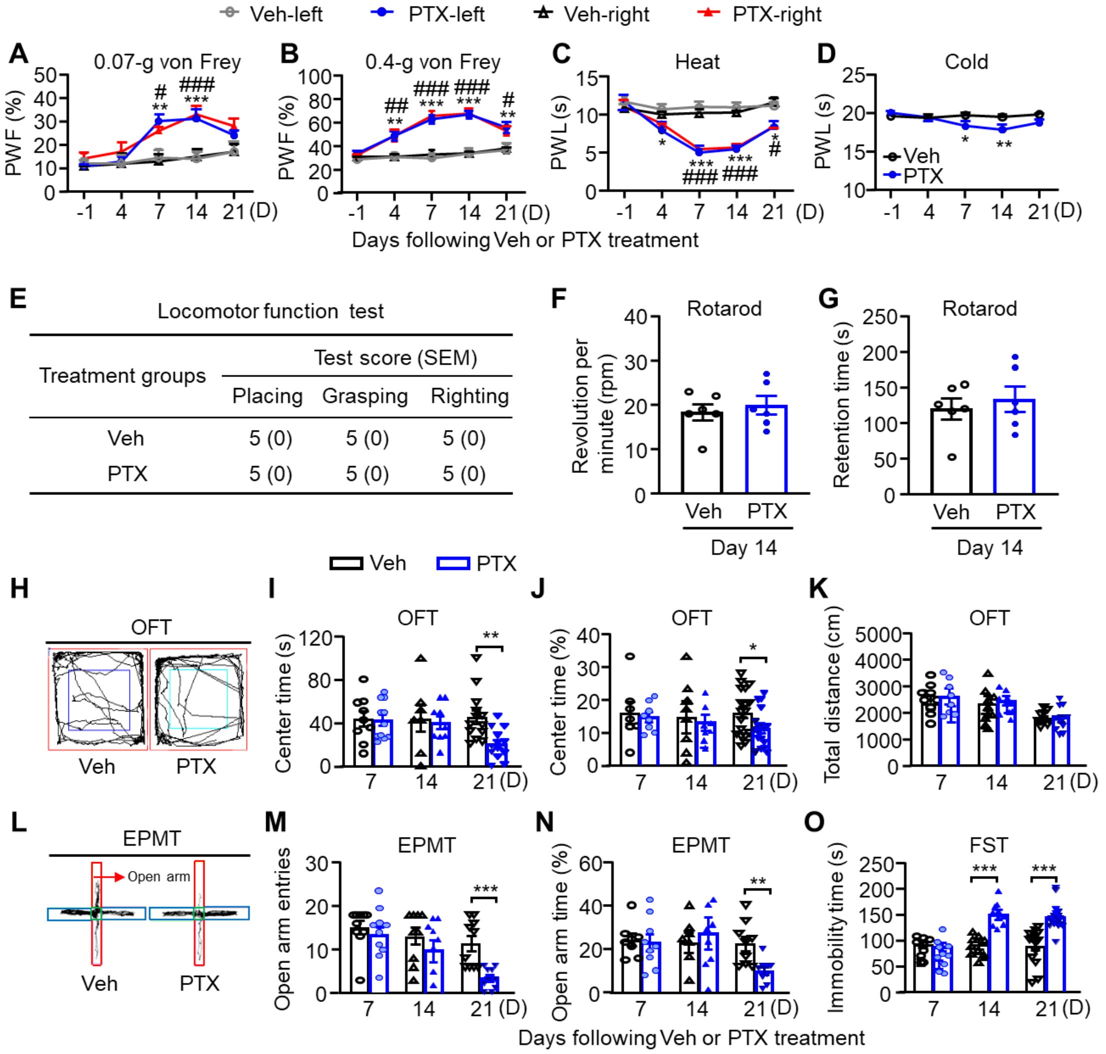

Figure S1 Paclitaxel (PTX) induces pain hypersensitivity, as well as anxiety-like and depression-like behaviors in male mice. A-D) The paw withdrawal responses to 0.07 g *von* Frey filament (A), 0.4 g *von* Frey filament (B), heat (C) and cold stimulation (D) following PTX or vehicle (Veh) treatment. n = 9-11. *P < 0.05, **P < 0.01, ***P < 0.001, *versus* the Veh group (left); $^{\#}P$ < 0.05, $^{\#\#}P$ < 0.01, $^{\#\#\#}P$ < 0.001, *versus* the Veh group (right), using two-way ANOVA followed by *post hoc* Tukey test. E) Locomotor function was assessed by scoring placing, grasping, and righting reflexes. Five trials for each reflex. n = 6. F, G) The rotational speed (revolutions per minute, rpm) and the retention time in the rotarod test. n = 6. H-K) Schematic traces of the open field test (OFT) (H) and the time spent in the center (I) and the percentage of time spent in the center (J), and the total distance traveled in the center (K) in the OFT. L-N) Schematic traces of the elevated plus-maze test (EPMT) (L) and the number of entries into the open arms (M) and the percentage of time (N) spent in the open arms in the EPMT following PTX treatment. O) The immobility time in the forced swim test (FST) following PTX treatment. n = 9-11. *P < 0.05, **P < 0.01, ***P < 0.001, *versus* the Veh group, using two-tailed unpaired t-tests.

Figure S2

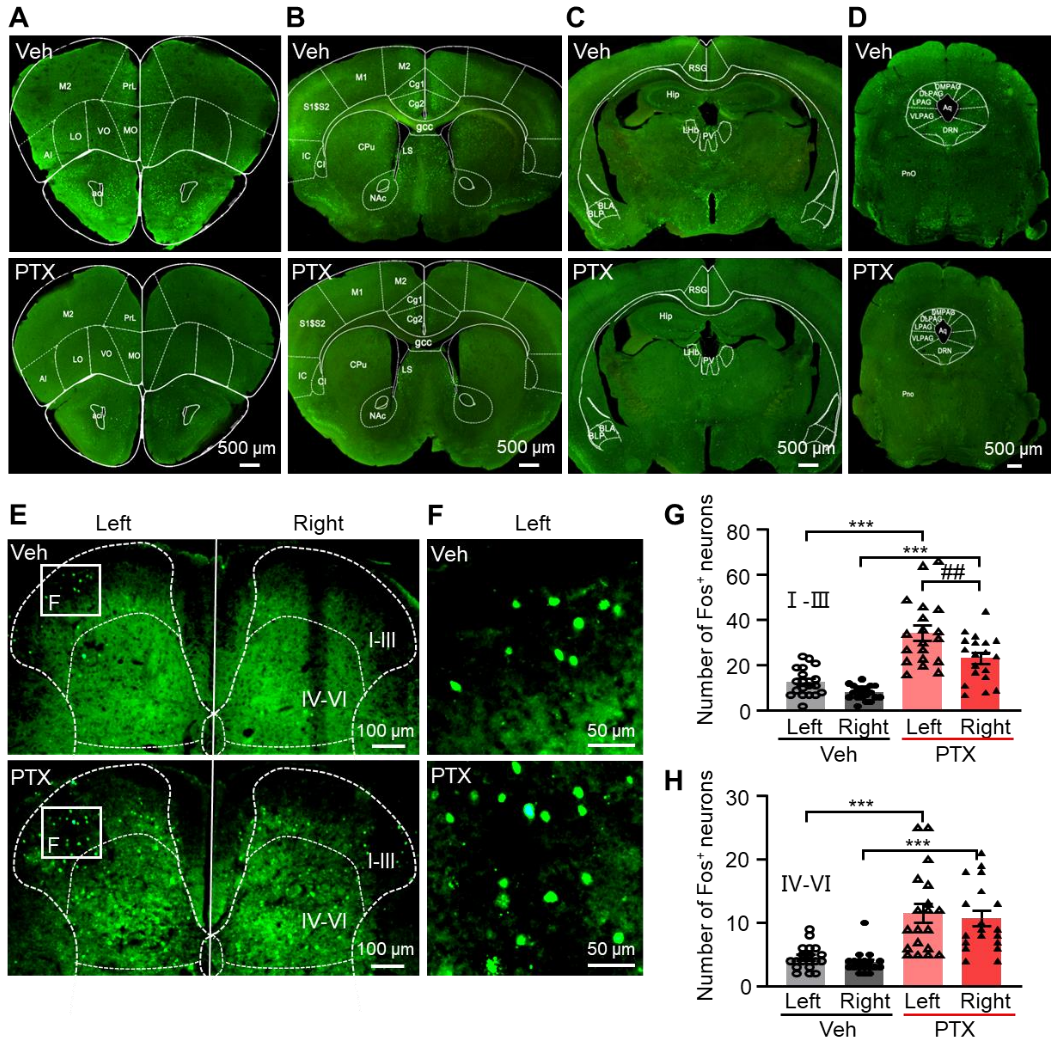

Figure S2 Fos expression in neurons across various brain regions and the lumbar spinal dorsal horn in mice treated with vehicle or paclitaxel (PTX). A) The medial prefrontal cortex (mPFC) (coordinates: anterior-posterior (AP) = + 2.0 mm, medial-lateral (ML) = ± 0.4 mm, dorsal-ventral (DV) = − 2.3 mm from the bregma); the ventrolateral orbital cortex (VLO) (coordinates: AP = + 2.4 mm, ML = ± 1.0 mm, DV = − 2.4 mm). B) The insular cortex (IC) (coordinates: AP = − 0.35 mm, ML = ± 4.0 mm, DV = − 3.8 mm); the anterior cingulate cortex (ACC) (coordinates: AP = + 1.0 mm, ML = ± 0.25 mm, DV = − 1.9 mm); the nucleus accumbens core (AcbC) (coordinates: AP = + 1.5 mm, ML = ± 0.75 mm, DV = − 3.9 mm). C) The basolateral amygdala (BLA) (coordinates: AP = − 1.3 mm, ML = ± 3.4 mm, DV = − 4.9 mm). D) The periaqueductal gray (PAG) (coordinates: AP = − 4.6 mm, ML = ± 0.5 mm, DV = − 3.3 mm); the dorsal raphe nucleus (DRN) (coordinates: AP = − 4.36 mm, ML = ± 0 mm, DV = − 3.0 mm). *n* = 3. Scale bar: 500 μm. E-G) Representative images (E, F) and histograms (G, H) show the increased number of Fos^{+} neurons in both superficial (I-III) (G) and deep (IV-VI) (H) laminae of the lumbar 4-5 spinal dorsal horn on day 14 following the initial injection of vehicle or PTX. The left paw was brushed for 1 minute, two hours before perfusion. Scale bar: 100 μm; 50 μm. ***$P < 0.001$, *versus* the Veh group (left or right); ##$P < 0.01$, *versus* the right side; using one-way ANOVA followed by *post hoc* Tukey test.

Figure S3

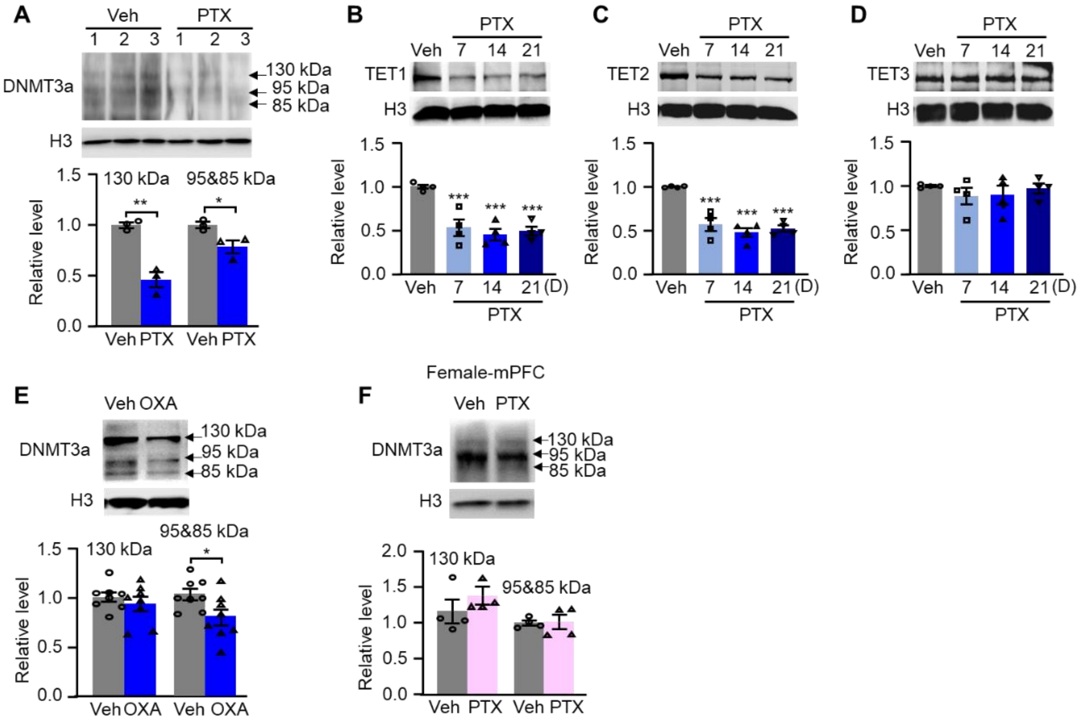

Figure S3 Paclitaxel (PTX) induces changes in the expression of DNMT3a protein and ten-eleven translocation (TET) methylcytosine dioxygenases in the mPFC. A) Expression of DNMT3a protein in the mPFC of male mice following treatment with PTX or vehicle (Veh). $n = 3$. $^{*}P < 0.05$, $^{**}P < 0.01$, *versus* the Veh group, using two-tailed unpaired *t*-tests. B-D) Protein expression levels of the DNA demethylases TET1 (B), TET2 (C), and TET3 (D) in the mPFC following treatment with PTX or Veh. $n = 4$. $^{***}P < 0.001$, *versus* the Veh group, using one-way ANOVA followed by *post hoc* Tukey test. E) The expression of DNMT3a protein in the mPFC of male mice following treatment with oxaliplatin (OXA). $n = 8$. $^{*}P < 0.05$, *versus* the Veh group, using two-tailed unpaired *t*-tests. $n = 4$. F) The expression of DNMT3a protein in the mPFC of female mice following treatment with PTX. n = 4. Representative western blots (top panels) and a summary of densitometric analysis (bottom graphs).

Figure S4

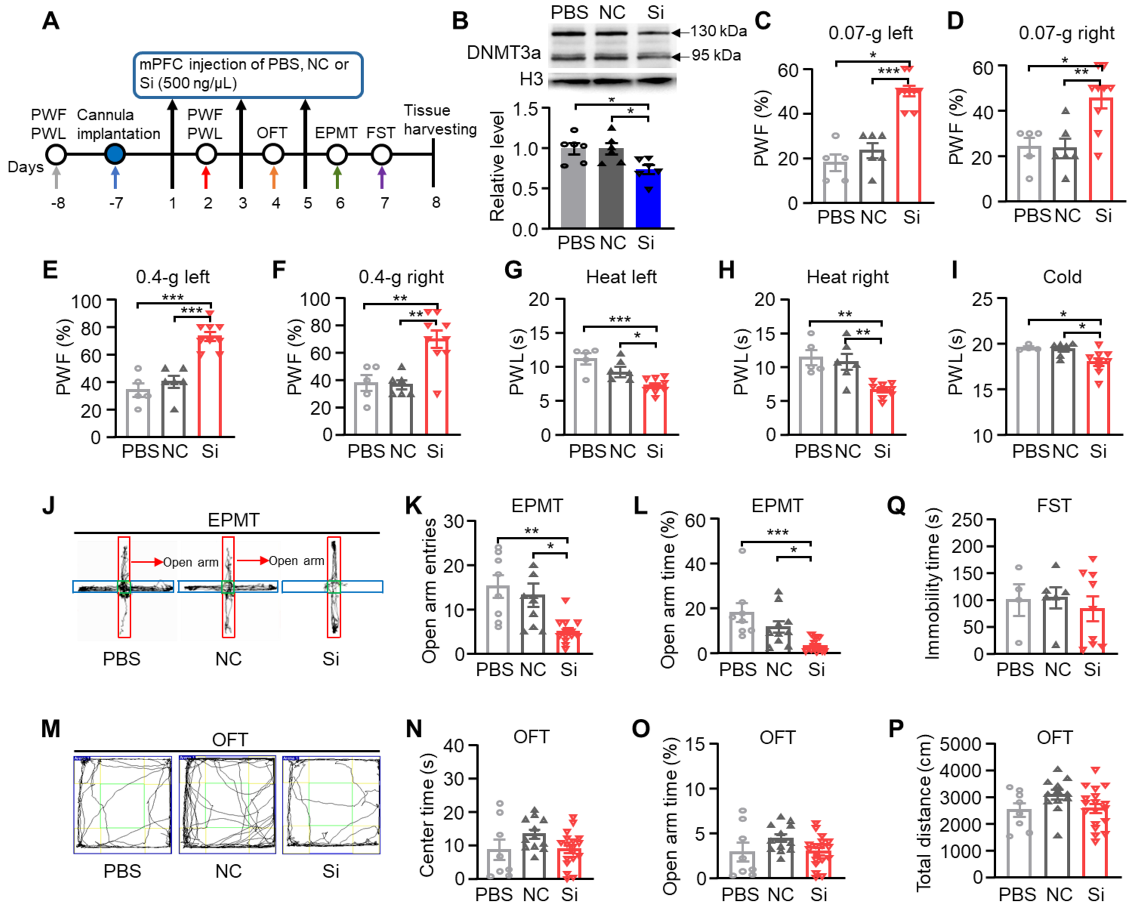

Figure S4 Knockdown of DNMT3a in the mPFC leads to pain hypersensitivity and anxiety-like behavior. A) A schematic diagram illustrating the administration of drugs and the sequence of behavioral tests. B) Reduction of DNMT3a protein levels in the mPFC following *Dnmt3a* siRNA administration. Representative western blots (top panels) and a summary of densitometric analysis (bottom graphs). $n = 6$. $^*P < 0.05$, using one-way ANOVA followed by *post hoc* Tukey test. C-I) Paw withdrawal responses to 0.07 g *von* Frey filament (C and D), 0.4 g *von* Frey filament (E and F), heat (G and H) and cold stimulation (I) following treatment. $n = 4$-9. $^*P < 0.05$, $^{**}P < 0.01$, $^{***}P < 0.001$, *versus* the control group using one-way ANOVA followed by *post hoc* Tukey test. J-L) Schematic traces of the elevated plus-maze test (EPMT) (J), the number of entries into the open arms (K), and the percentage of time (L) spent in the open arms in the EPMT following treatment. M-P) Schematic traces of the open field test (OFT) (M), the time spent in the center (N), the percentage of time spent in the center (O), and the total distance traveled in the center (P) in the OFT following treatment. Q) The immobility time in the forced swim test (FST) following treatment. $n = 8$-13. $^*P < 0.05$, $^{**}P < 0.01$, $^{***}P < 0.001$, *versus* the PBS or NC group, using one-way ANOVA followed by *post hoc* Tukey test. Si: *Dnmt3a* siRNA; NC: negative control siRNA; PBS: phosphate-buffered saline.

Figure S5

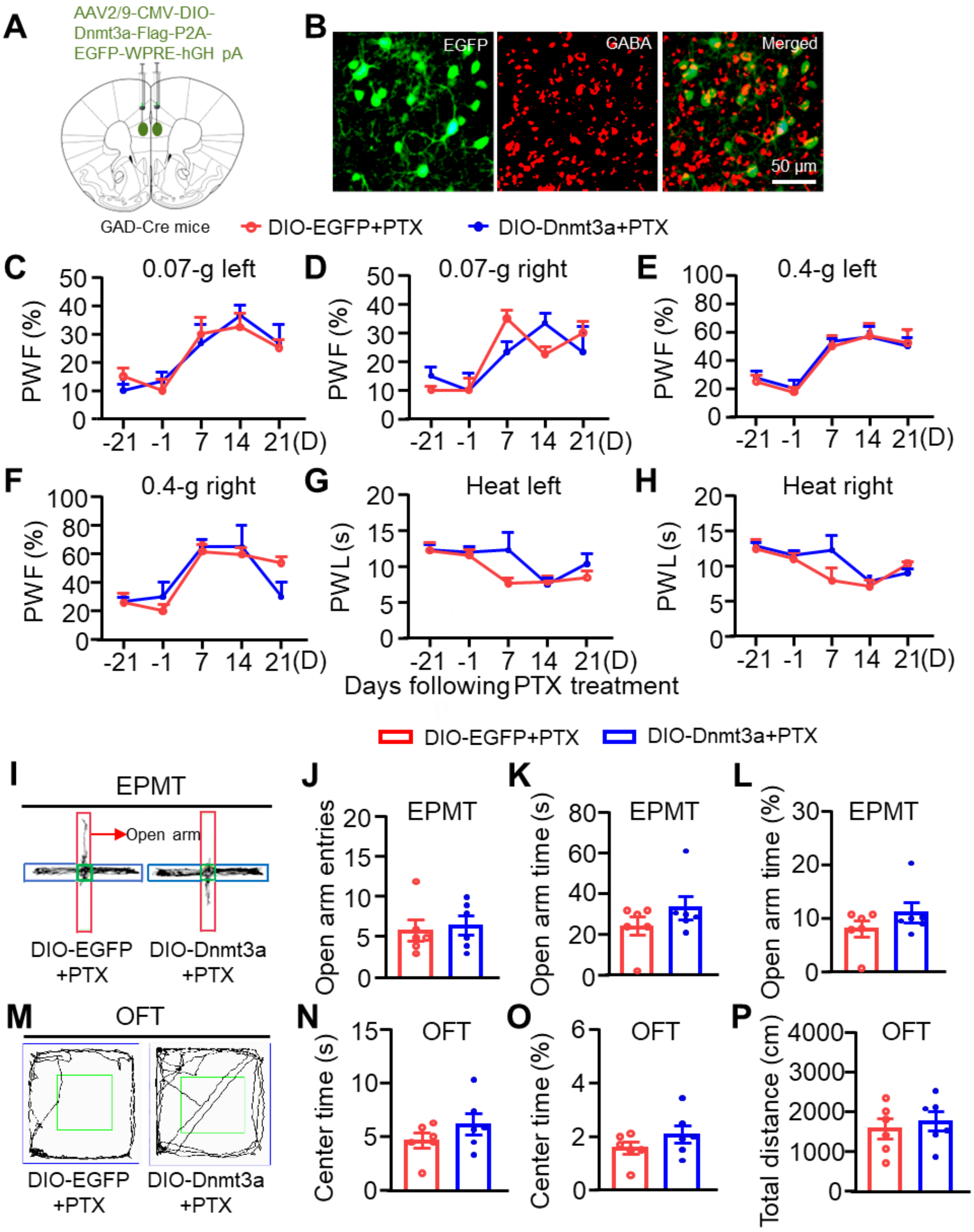

Figure S5 Overexpression of DNMT3a in GABAergic neurons of the mPFC does not affect paclitaxel (PTX)-induced pain hypersensitivity or anxiety-like behavior. A) Schematic diagram of mPFC injected with AAV-DIO-*Dnmt3a* in GAD-Cre mice. B) Colocalization of EGFP (green) with GABA (red) in the mPFC across three mice. Scale bar: 50 μm. C-H) PTX was administered three weeks after the microinjection of AAV-DIO-EGFP or AAV-DIO-*Dnmt3a* into the mPFC, after which behavioral testing was conducted. Paw withdrawal responses to 0.07 g *von* Frey filament (C, D), 0.4 g *von* Frey filament (E, F) and heat stimulation (G, H) following treatment with PTX. *n* = 6. I-L) Schematic traces of the elevated plus-maze test (EPMT) (I) and the number of entries into the open arms (J), the time (K) and the percentage of time (L) spent in the open arms in the EPMT. M-P) Schematic traces of the open field test (OFT) (M) and the time spent in the center (N) and the percentage of time spent in the center (O), and the total distance traveled in the center (P) in the OFT. *n* = 6.

Figure S6

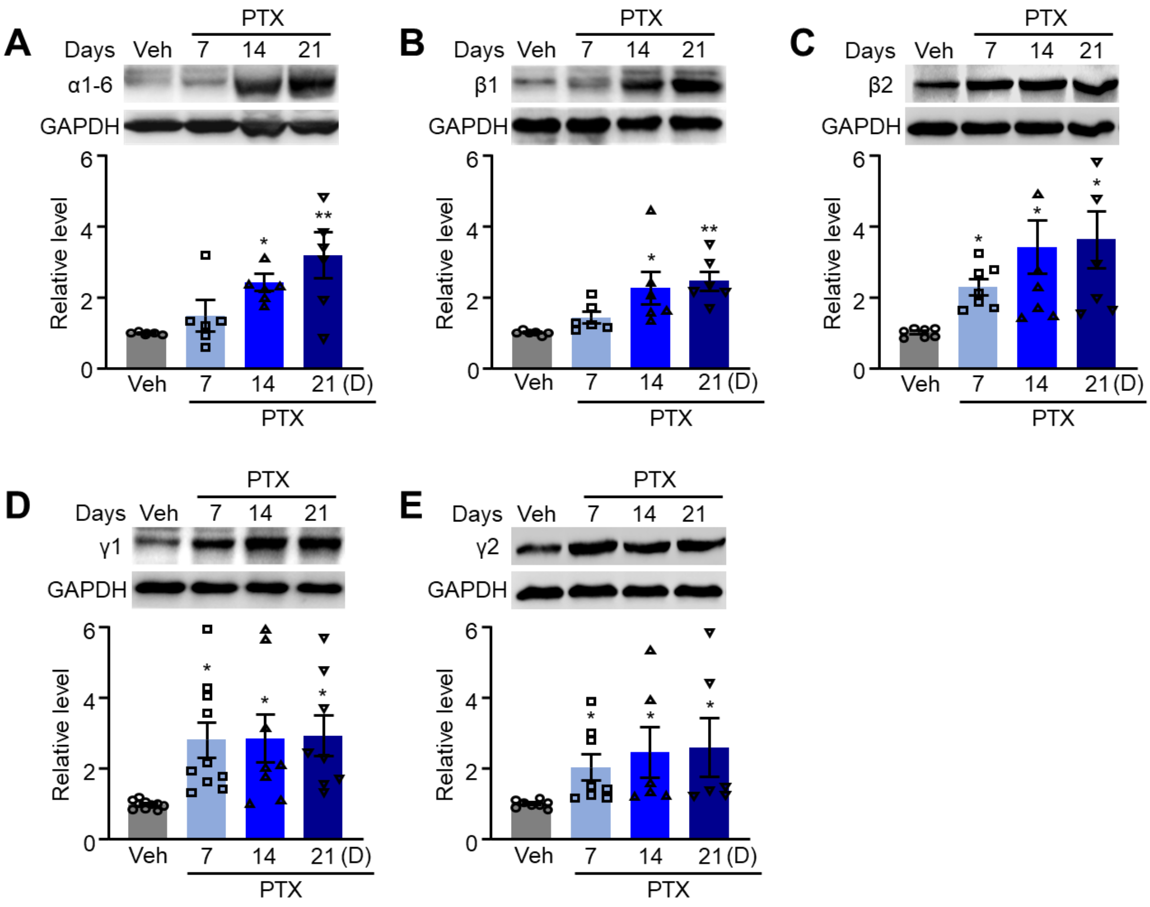

Figure S6 Paclitaxel (PTX) induces changes in the expression of $GABA_A$ receptor subunits in the mPFC following treatment. A-E) α1-6 subunits (A), β1 subunit (B), β2 subunit (C), γ1 subunit (D) and γ2 subunit (E). Representative western blots (top panels) and a summary of densitometric analysis (bottom graphs). n = 6-10. *$P < 0.05$, **$P < 0.01$, *versus* the vehicle (Veh)-treated group, using one-way ANOVA followed by *post hoc* Tukey test.

Figure S7

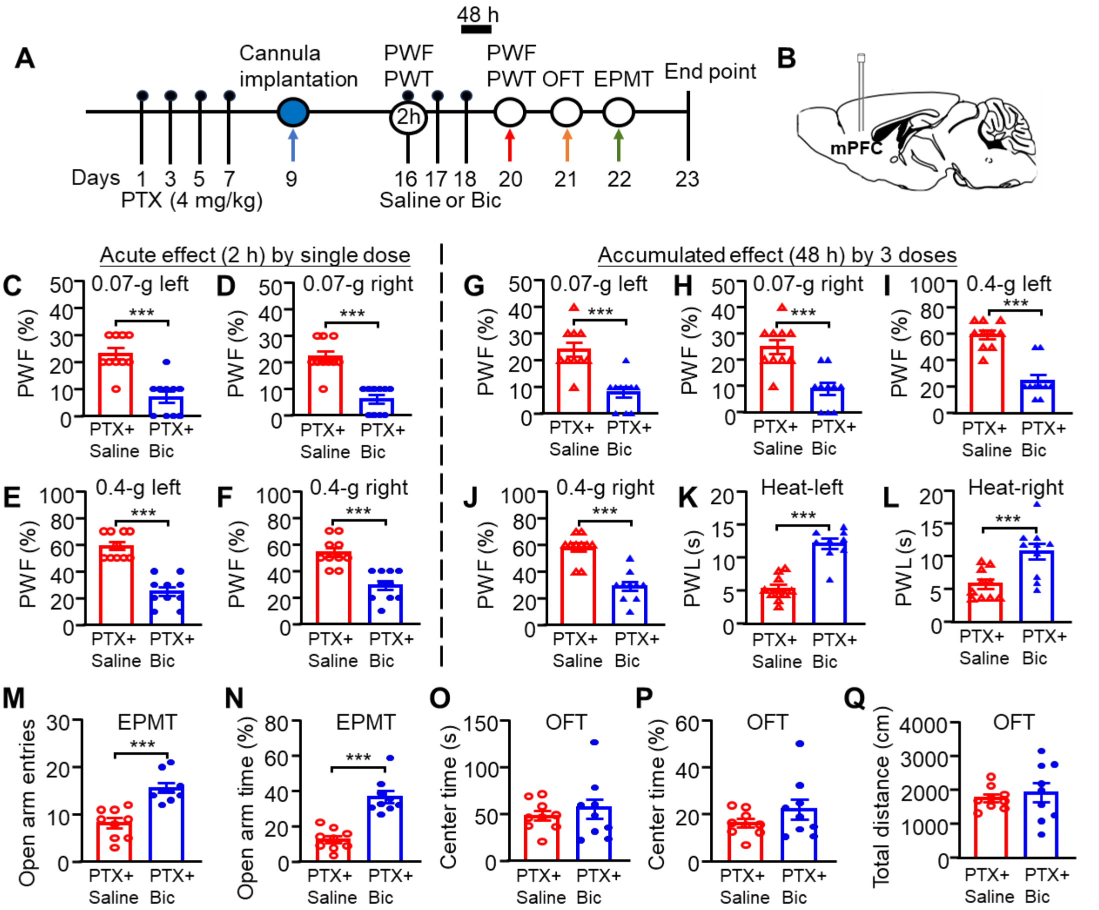

Figure S7 Suppression of $GABA_A$ receptor activity in the mPFC reverses pain hypersensitivity and anxiety-like behavior induced by paclitaxel (PTX). A) Timelines for drug administration, cannula implantation, and behavioral testing. B) A schematic of cannula implantation into the mPFC. C-F) Paw withdrawal responses to the 0.07 g von Frey filament (C, D) and the 0.4 g von Frey filament (E, F) following mPFC injection of physiological saline or bicuculline (Bic, 100 ng) within 2 hours after intraperitoneal PTX injection. G-L) Paw withdrawal responses to the 0.07 g von Frey filament (G, H), the 0.4 g von Frey filament (I, J), and heat stimulation (K, L) following mPFC injection of saline or Bic for three consecutive days, from day 16 to day 18, after PTX treatment. M-Q) The entries (M) and the percentage of time (N) into the open arm in the elevated plus-maze test (EPMT) following mPFC injection of saline or Bic for three consecutive days after PTX treatment. The time (O), the percentage of time (P) and total distance traveled (Q) in the central area in the open field test (OFT) following mPFC injection of saline or Bic for three consecutive days after PTX treatment. n = 10. ***P < 0.001, *versus* the PTX-treated with the saline group, using two-tailed unpaired t-tests.

Figure S8

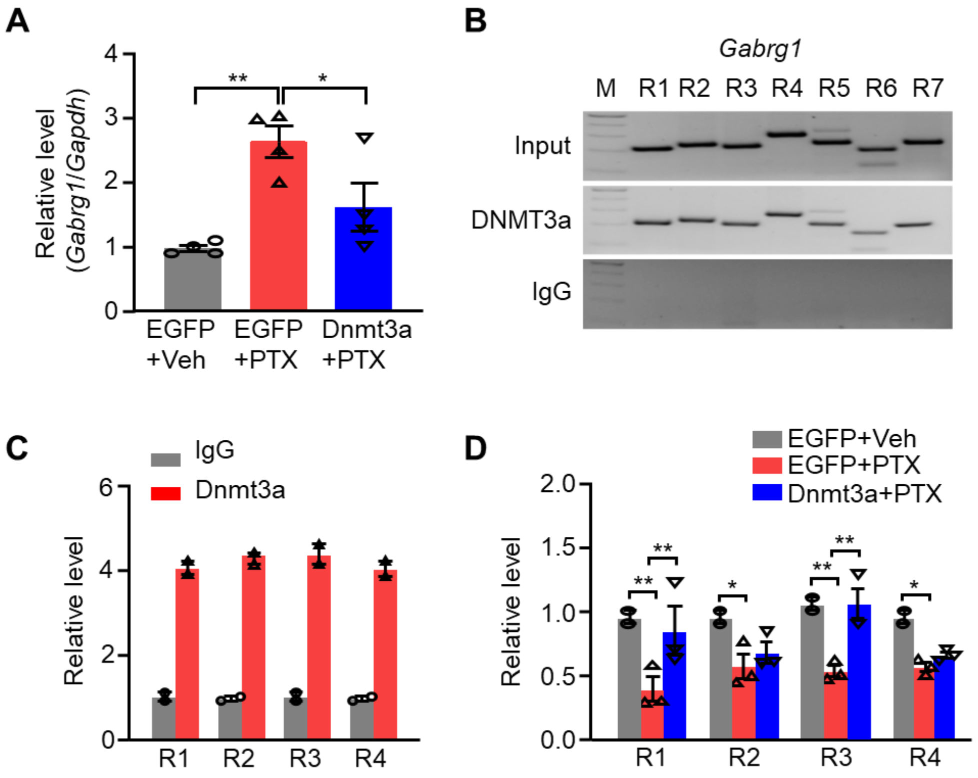

Figure S8 DNMT3a regulates the transcriptional expression of the *Gabrg1* gene in the mPFC. A) *Gabrg1* mRNA levels in the mPFC following treatment with paclitaxel (PTX) or vehicle (Veh). $n = 4$. *$P < 0.05$, **$P < 0.01$, using one-way ANOVA followed by *post hoc* Tukey test. B-C) Seven regions from the promoter and 5'-end untranslated regions of the *Gabrg1* gene were immunoprecipitated by the rabbit anti-DNMT3a. Input, total purified fragments. IgG, negative control. M, ladder marker. $n = 2$ biological replicates/6 mice/group. D) The binding activity of DNMT3a to the R1 (-1002 bp to -789 bp) and R3 (-598 bp to -381 bp) regions, the R2 (-810 bp to -577 bp) and R4 (-402 bp to -113 bp) regions, within the *Gabrg1* gene is restored by the overexpression of DNMT3a in the mPFC. $n = 3$ biological replicates/9 mice/group. *$P < 0.05$, **$P < 0.01$, using one-way ANOVA followed by *post hoc* Tukey test.

Table S1. The information of antibodies, viruses, and chemicals.

REAGENT or RESOURCE	SOURCE	IDENTIFIER
Antibodies		
DNMT3A (E9P2F) Rabbit mAb	Cell signaling Technology	Cat# 49768; RRID: AB_2799365
DNMT3A Antibody	Cell signaling Technology	Cat# 2160; RRID: AB_2263617
DNMT3B (E4I4O) Rabbit mAb (Mouse Specific)	Cell signaling Technology	Cat #48488; RRID: AB_2799339
DNMT1 (D63A6) XP® Rabbit mAb	Cell signaling Technology	Cat# 5032; RRID: AB_10548197
TET1 Rabbit Polyclonal Antibody	Beyotime	Cat# AF8118; RRID: AB_3066763
TET2 Rabbit Polyclonal Antibody	Beyotime	Cat# AF8119; RRID: AB_3066781
TET3 Rabbit Polyclonal Antibody	Beyotime	Cat# AF8121; RRID: AB_3066782
$GABA_A$ Rα1-6 (E-8)	Santa Cruz Biotechnology	Cat# sc-376282; RRID: AB_10988210
Anti-GABRB1 antibody [N96/55]	Abcam	Cat# ab93612; RRID: AB_10565043
Rabbit Anti-GABRB2 Polyclonal Antibody	Bioss	Cat# bs-12065R; RRID: AB_3066681
Anti-GABA(A) γ1 Receptor Antibody	alomone labs	Cat# AGA-016; RRID: AB_2340941
Rabbit Anti-GABAA Receptor gamma2 Polyclonal Antibody	Bioss	Cat# bs-4112R; RRID: AB_11075070
Anti-NMDAR2A/GRIN2A Antibody	Boster	Cat# BA0613; RRID: AB_3083488
Anti-NMDAR2B/GRIN2B Antibody	Boster	Cat# BA0614-2; RRID: AB_3083489
Anti-GAPDH Antibody	Boster Biological Technology	Cat# BA2913; RRID: AB_2560936
Histone H3 Antibody	Cell Signaling Technology	Cat# 9715; RRID: AB_331563
Anti-c-Fos antibody	Abcam	Cat# ab222699; RRID: AB_2891049
CaMKIIα (6G9) Mouse mAb	Cell signaling Technology	Cat# 50049S; RRID: AB_2721906
CaMKIIα/δ Polyclonal Antibody	Immunoway	Cat# YT0623; RRID: AB_3083585
Anti-GABA Antibody	Sigma-Aldrich	Cat# A2052; RRID: AB_477652
Goat Anti-Glutamate Transporter, Neuronal (EAAC1) Polyclonal antibody	Upstate/EMD Millipore	Cat# AB1520; RRID: AB_90732
HRP-conjugated goat anti-rabbit IgG	Sigma-Aldrich	Cat# AP132P; RRID: AB_3083577
HRP-conjugated goat anti-mouse IgG	DIYIBio	Cat# DY60203; RRID: AB_3083582
CY3-conjugated goat anti-rabbit IgG	Upstate/EMD Millipore	Cat# AP132C; RRID: AB_92489
CY3-conjugated rabbit anti-goat IgG	Upstate/EMD Millipore	Cat# AP106C; RRID: AB_92245
FITC- conjugated donkey anti-mouse IgG	Upstate/EMD Millipore	Cat# AP192F; RRID: AB_92643
Alexa Fluor® 488-conjugated donkey anti-rabbit IgG	Abcam	Cat# ab150073; RRID: AB_2636877
Alexa Fluor® 488-conjugated goat anti-mouse IgG	Abcam	Cat# ab150133; RRID: AB_2576208
Virus Strains		
rAAV-CMV-EGFP-WPRE-hGH pA, AAV2/9	BrainVTA	Cat# PT1316
rAAV-CMV-DNMT3a-Flag-P2A-EGFP-WPRE-hGH pA, AAV2/9	BrainVTA	Cat# PT1759

rAAV-CMV-DIO-EGFP-WPRE-hGH pA, AAV2/9	BrainVTA	Cat# PT0310
rAAV-CMV-DIO-DNMT3a-Flag-P2A-EGFP-WPRE-hGH pA, AAV2/9	BrainVTA	Cat# PT6034
Chemicals		
Paclitaxel	MedChemExpress	Cat# HY-B0015
Oxaliplatin	MedChemExpress	Cat# HY-17371
Cremophor EL	Sigma-Aldrich	Cat# C5135
Bicuculline	MedChemExpress	Cat# HY-N0219/CS-5493
Picrotoxin	Sigma-Aldrich	Cat# R284556
6-cyano-7-nitroquinoxaline-2,3-dione	Tocris	Cat# 0190

Table S2. All primers.

Names	Sequences	Names	Sequences
Real-time		ChIP-PCR	
RT-PCR		*Gabrb1F1-F*	5'-AGTCAGGTCTCAAGAGGTAATT-3'
Dnmt3a-F	5'-GCCAAGAAACCCAGAAAGAGC-3'	*Gabrb1F1-R*	5'-TGAAAACCAGTCATTCTCTCG-3'
Dnmt3a-R	5'-GCACATGCCTCCAATGAAGA-3'	*Gabrb1F2-F*	5'-CGAGAGAATGACTGGTTTTCA-3'
Dnmt3b-F	5'-CAGCCTTCTGAATTACACGCA-3'	*Gabrb1F2-R*	5'-ACAGGCTGCGTCCTGGATTT-3'
Dnmt3b-R	5'-TCCCATTGCTATGTCGGGTT-3'	*Gabrb1F3.1-F*	5'-TCCCCACCGAACTCACCAA-3'
Dnmt1-F	5'-AGTCGGACAGTGACACCCTTT-3'	*Gabrb1F3.1-R*	5'-TGTTGCGAAGATGGTTTCTGT-3'
Dnmt1-R	5'-TGTGTCTACAACTCTGCGTTTCT-3'	*Gabrb1F3.2-F*	5'-TCCCCACCGAACTCACCAA-3'
mGabrb1-F	5'-CCCGTGATGGTTGCTATGGT-3'	*Gabrb1F3.2-R*	5'-GCCACTCTGCCCTCTCCT-3'
mGabrb1-R	5'-TATGCTGGCGACATCGATCC-3'	*Gabrb1F4-F*	5'-ACAGAAACCATCTTCGCAACA-3'
mGabrg1-F	5'-AAAAACCACCAGAGGCAGGA-3'	*Gabrb1F4-R*	5'-TGAGCAGCACTTGCAAGCTT-3'
mGabrg1-R	5'-TCATCTTCCCCTTGTGGCAA-3'	*Gabrb1F5-F*	5'-AGGAGAGGGCAGAGTGGC-3'
Gapdh-F	5'-TCGGTGTGAACGGATTTGGC-3'	*Gabrg1F4-F*	5'-ACTGATGAGAGATGTACATTGC-3'
Gapdh-R	5'-TCCCATTCTCGGCCTTGACT-3'	*Gabrg1F4-R*	5'-TGGACAGAGCCCAATTGACA-3'
		Gabrg1F5-F	5'-TGTCAATTGGGCTCTGTCCA-3'
Bisulfite		*Gabrg1F5-R*	5'-CCTCTTCTCAGCCCCAGT-3'
Sequencing		*Gabrg1F6-F*	5'-ACTGGGGCTGAGAAGAGG-3'
Gabrb1F1-F	5'-TTAGTGTGGGGGAGGTTAAG-3'	*Gabrg1F6-R*	5'-CTCCCAGCAGAACTGAGCTA-3'
Gabrb1F1-R	5'-CCTCACCTACAAAAATAAAAACCAATCATT-3'	*Gabrg1F7-F*	5'-TAGCTCAGTTCTGCTGGGAG-3'
		Gabrg1F7-R	5'-AATATCAACCTCACTCCTCTAG-3'
Gabrb1F1-S	5'-GGGGAGGTTAAGTTT-3'		

F: Forward. R: Reverse.

Original western blots

Figure 1E

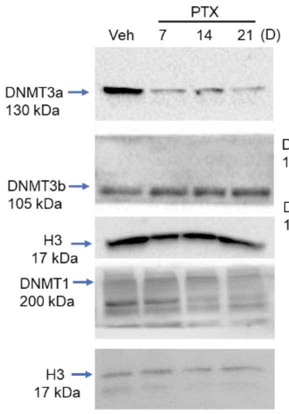

Figure 1F

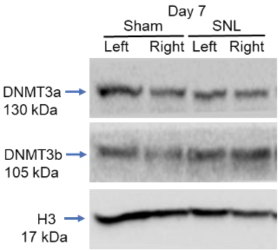

Figure 1G

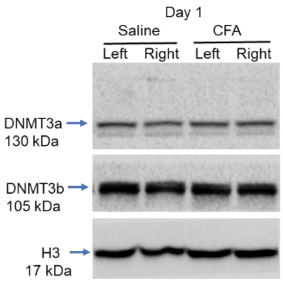

Figure 2E

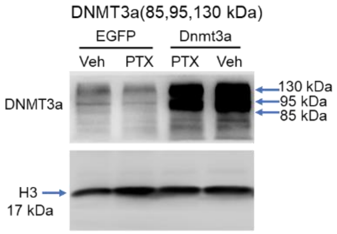

Figure 6A

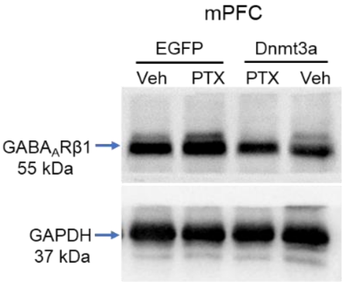

Figure 6B

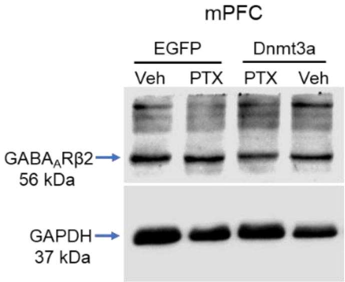

Figure 6C

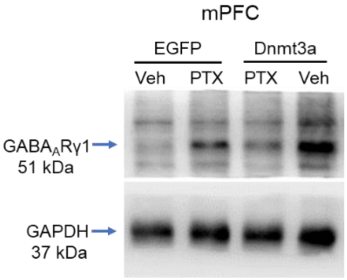

Figure 6D

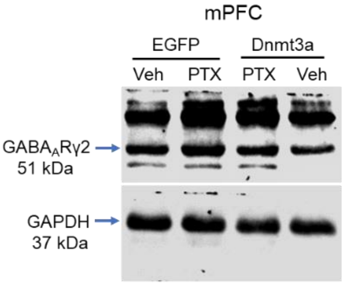

Figure 6E

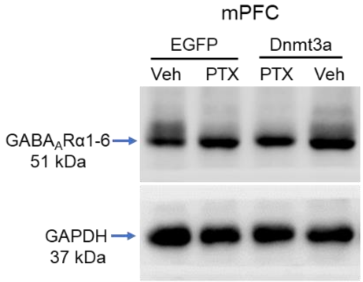

Figure 6G

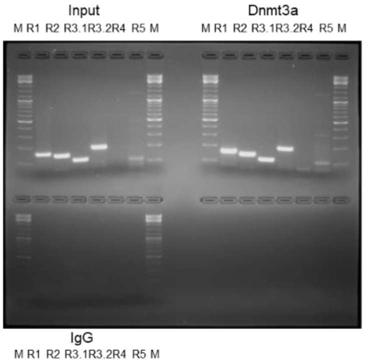

Supplementary Figure 3A

mPFC

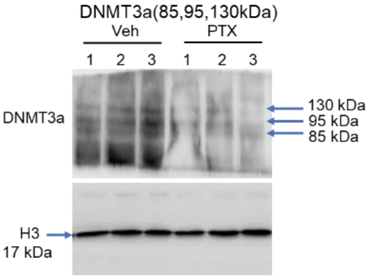

Supplementary Figure 3B

mPFC

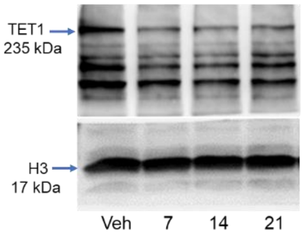

Supplementary Figure 3C

mPFC

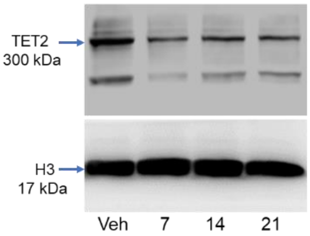

Supplementary Figure 3D

mPFC

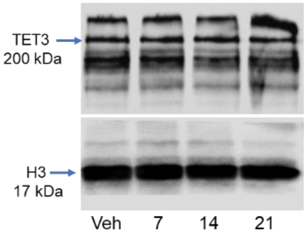

Supplementary Figure 3E

mPFC

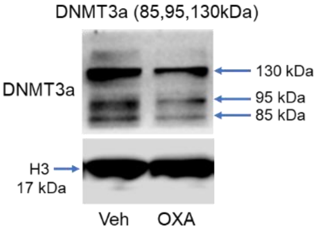

Supplementary Figure 3F

mPFC

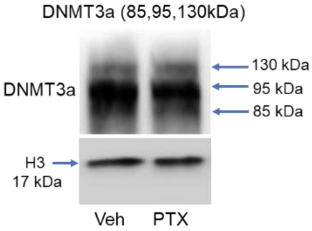

Supplementary Figure 4A

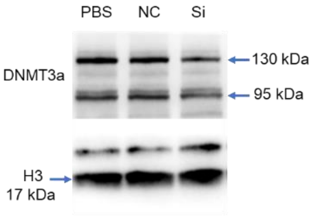

Supplementary Figure 6A

mPFC

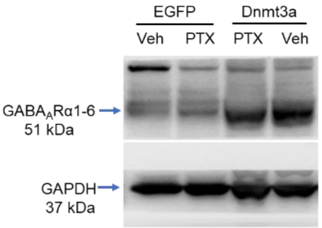

Supplementary Figure 6B

mPFC

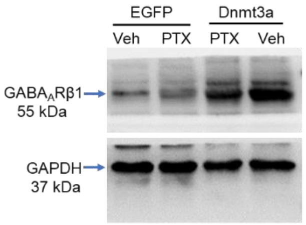

Supplementary Figure 6C

mPFC

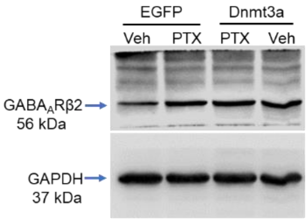

Supplementary Figure 6D

mPFC

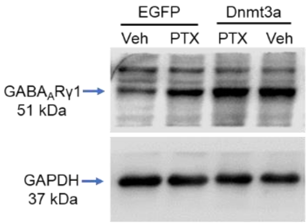

Supplementary Figure 6E

mPFC

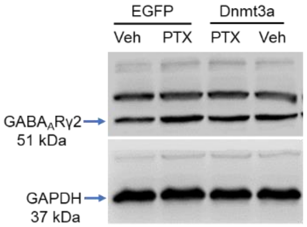

Supplementary Figure 8B

Input

M R1 R2 R3 R4 R5 R6 R7 M

Dnmt3a

M R1 R2 R3 R4 R5 R6 R7 M

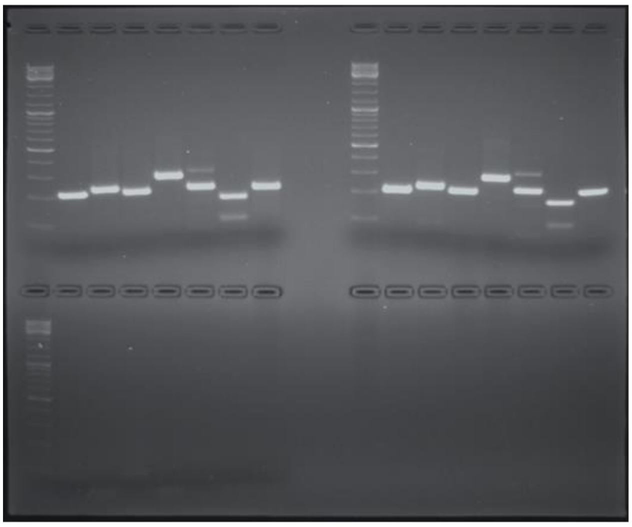

IgG

M R1 R2 R3 R4 R5 R6 R7 M
